# Top 10 Research Lessons Learned From a Digital Child-Rearing Program in Low- and Middle-Income Countries: Multicase Study

**DOI:** 10.2196/65705

**Published:** 2025-07-29

**Authors:** Haley M LaMonica, Victoria Loblay, Adam Poulsen, Gabrielle Hindmarsh, Mafruha Alam, Mahalakshmi Ekambareshwar, Qaisar Khan, Yun J C Song, Jacob J Crouse, Chloe Wilson, Madelaine Sweeney-Nash, Olivia Iannelli, Aila Naderbagi, Iqthyer U Zahed, Adam Yoon, Mujahid Torwali, Jakelin Troy, Ian B Hickie

**Affiliations:** 1 Brain and Mind Centre The University of Sydney Camperdown Australia; 2 Faculty of Arts and Science The University of Sydney Sydney Australia

**Keywords:** child development, parenting program, digital technology, global health, participatory research, stakeholder participation, mobile app, process evaluation, low- and middle-income countries

## Abstract

**Background:**

Extensive literature highlights the effectiveness of parenting programs for early childhood and parental outcomes globally. Increasing evidence shows that digital parenting programs are as effective as those delivered in person and that digital delivery is acceptable to parents. However, parenting programs cannot be one-size-fits-all but rather need to be developed, adapted, and refined to account for the context, culture, attitudes, behaviors, and expectations of the intended target audience.

**Objective:**

This study aimed to identify the key research lessons learned from Minderoo Foundation’s Thrive by Five International Program, a large-scale digital and nondigital child-rearing program, including how they relate to research and development (R&D) processes and sociocultural context. The “core elements” of R&D identified in the Medical Research Council’s framework for developing and evaluating complex interventions served as a guide to synthesize the data from this 3-year (2021-2024) program.

**Methods:**

We used a multicase study design to build a deep understanding of the program, including how it varied across and was influenced by diverse sociocultural and contextual factors across 10 low- and middle-income countries. Data analysis for each case occurred over 3 phases, including qualitative data analysis and reporting, data synthesis to inform the transfer of learnings to the program, and, finally, a secondary analysis relating to program theory, stakeholder engagement, and the refinement of the program as they related to, interacted with, and were influenced by context.

**Results:**

The analysis resulted in five themes: (1) the role and value of partnerships, including the importance of selecting partners with strong and broad networks; (2) building collaborative practice with partners, which identifies strategies to foster collaboration; (3) honing a target audience, which emphasizes the importance of identifying the end user at the start of R&D; (4) navigating the digital landscape, including the use of context-specific dissemination strategies; and (5) managing linguistic diversity and translation, including the value of embedding a translator on the project team. Learnings regarding context and cultural diversity were integrated throughout the results.

**Conclusions:**

Digital parenting programs must be appropriate for and accessible to the target audience, aligned with information and communications technology infrastructure and policies, and fill a need in the digital health marketplace. When this is not feasible, a multichannel approach to dissemination using digital and nondigital strategies is necessary. While likely to increase project complexity, cross-sectoral partnerships, including with government bodies, are likely to broaden the program’s reach. To facilitate digital parenting projects, it is critical that sufficient time be allocated to build meaningful collaborative partnerships centered on respect, cultural understanding, and open communication and grounded by a shared vision.

## Introduction

### Parenting Programs

The United Nations Sustainable Development Goal 4.2 aims to ensure that all children have access to quality early childhood development, care, and preprimary education so that they are ready for primary education by 2030 [1]. Globally, parenting programs have long been viewed as a critical strategy to help ensure children reach their full developmental potential [2-4], with robust evidence of effectiveness for both early childhood and parental outcomes [5-10]. Within this broader evidence base, a large systematic review of 111 studies found that parenting interventions have a larger effect in low- and middle-income countries (LMICs) relative to high-income countries (HICs), including for parenting practices and child cognitive, language, and motor development outcomes [8]. Notably, in both HICs and LMICs, interventions that promote responsive caregiving (ie, a caregiver’s timely attention to, understanding of, and responsiveness to children’s movements and verbal and nonverbal signals) [11] have been shown to be more effective in improving parent-child interactions, parental knowledge, and parenting practices relative to those programs without an emphasis on responsive care [8]. In turn, responsive and nurturing care as well as safety provided by parents and families serve as buffers to adversity in early childhood, promoting health and well-being that extends into adolescence and adulthood [12].

### Digital Delivery of Parenting Programs

To drive progress toward the United Nations Sustainable Development Goals, the United Nations General Assembly specifically highlighted the opportunity to capitalize on the spread of information and communications technology (ICT) to accelerate human progress worldwide [1]. The COVID-19 pandemic resulted in the rapid adoption of digital technologies by governments, businesses, schools, and individuals, including a 15% increase in the use of the internet in LMICs during 2020 [13]. Acknowledging that a digital divide exists and persists globally, particularly among women [14], Indigenous communities [15], and individuals living in low-income countries [16], ICT infrastructure clearly has tremendous potential to increase accessibility to parenting programs and supports for families and communities. To that end, ICT has been used to flexibly deliver professional- and peer-led support, psychoeducation via websites and mobile apps, and self-directed web-based programs to support parents and families [17].

Importantly, evidence from HICs suggests that parenting programs delivered digitally can achieve the same level of effectiveness as those delivered in-person. For example, 10 of 12 studies in a meta-analytic review found online parenting programs significantly improved children’s emotional and behavioral functioning, with effect sizes (Hedges *g*) ranging from –0.22 to –0.32 [18]. Moreover, a recent systematic review found that online parenting programs were noninferior compared to in-person programs for improving parenting practices and reducing mental health problems for both children and parents [19]. There was also a trend toward greater parental satisfaction with online parenting programs, noting this result did not reach statistical significance [19]. This finding aligns with previous research indicating parents’ preference for self-administered parenting programs, such as those delivered via the web or written materials [20,21], in part because these formats mitigate barriers related to accessibility, cost, travel requirements, and stigma [22]. Nevertheless, it is important to recognize that most research about the digital delivery of parenting programs has been conducted in HICs, which leaves many unanswered questions about the generalizability of these findings to LMICs.

### Iterative Research and Development Processes for Complex Interventions

Parenting programs are typically considered complex interventions, as they often draw on varied sources of information, incorporate a range of intersecting components, use different therapeutic strategies, and target multiple outcomes [23]. The Medical Research Council’s (MRC) framework for developing and evaluating complex interventions, hereafter referred to as the MRC framework, emphasizes an iterative approach to development, feasibility testing, evaluation, and implementation of complex interventions [24]. At each phase in this cycle, learnings inform what are referred to as “core elements” that underpin all aspects of the research and development (R&D) process, including the overarching program theory, contextual factors, stakeholder engagement, and intervention refinements. Importantly, the framework guides researchers in the exploration of broad and complex questions (eg, how interventions interact with and influence the context in which they are implemented) rather than narrow research questions that require more precise answers (eg, is the intervention more effective at reducing symptoms of depression relative to treatment as usual) [24]. Notably, to conduct successful complex intervention research, the MRC states that varied research perspectives and methodologies are required.

### Considering Sociocultural Context

The MRC framework explicitly recognizes the dynamic and bidirectional interactions between interventions and context [25]. In relation to parenting programs, some topics or themes, such as the promotion of responsive care, are critical to effectiveness across contexts [8]; however, a one-size-fits-all approach is neither feasible nor appropriate. It is vital that the evidence base underpinning parenting programs is not confined solely to research conducted in Western, educated, industrialized, rich, and democratic (WEIRD) contexts [26]. Conceptualizations of and beliefs about early childhood development and parenting vary markedly across cultures and geographic areas [27,28]. While there are culture-common influences on parenting (eg, the need to keep young children safe), culture-specific factors (eg, social competencies and values) fundamentally shape how children are raised [29]. Therefore, as parenting programs are iteratively developed and refined, it is critical to understand and account for the context, culture, attitudes, behaviors, and expectations of the intended target audience [30,31]. Parenting programs cannot advocate for a single *right* way to raise a child but rather need to be adapted for the sociocultural context in which they will be implemented or be flexible enough to be effective across diverse settings [32].

There is growing literature about best practice approaches to support the cultural adaptation of digital interventions, such as parenting programs, for specific audiences and contexts [33,34]. This may include surface-level adaptation, such as content translation into different languages or the use of recognizable clothing or places in images [35]. However, to prevent exclusion of some groups (eg, on the basis of ethnicity, language, or socioeconomic status), which potentially further widen health inequities [32], deep structure adaptation is likely more effective in promoting cultural sensitivity and responsiveness in R&D processes [35]. Qualitative research methods are often used to inform such deep structure adaptation strategies. Furthermore, reframing standardization to relate to intervention change processes and functions (eg, providing training to parents about responsive caregiving) as opposed to intervention components (eg, in-person workshops and workbook materials) enables clinical trials of complex interventions to be conducted more feasibly to establish high-quality evidence of effectiveness across varied contexts [36].

### Objectives

Case study methodologies have been shown to be an effective and richly descriptive way of evaluating complex interventions [37,38]. In this study, we aimed to identify the key research lessons learned from a large-scale digital and nondigital child-rearing program—Minderoo Foundation’s Thrive by Five International Program—drawing on research conducted across 10 LMICs in Africa, Central Asia, Southeast Asia, and Melanesia. Data used in this study included field notes and transcriptions from semistructured and conversational interviews and group-based workshops. The MRC framework guided the approach to synthesizing the data from this 3-year (2021-2024) project. Specifically, the multicase study aims to (1) identify critical learnings about Thrive by Five across the MRC framework’s core elements throughout the 4 phases of research, (2) explore how these learnings related to core R&D processes, and (3) examine how these may have varied based on sociocultural context.

Importantly, context in this study is conceptualized as a dynamic process that shifts in relation to social beliefs, practices, and behaviors that intersect with and are influenced by the digital health intervention [39]. In this way, context is viewed “as something that happens and as something that researchers do (verb)” [39], rather than a separate research component to be examined in isolation. Therefore, we have not focused on lessons that explore “context” as a separate entity, as we consider this to permeate all areas of learning. Importantly, collaboration with key stakeholders has been critical to developing an understanding of the complex and evolving nature of context in each case. Concepts usually associated with context such as “culture” or “education” often functioned as “boundary objects” [40] that facilitated shared understandings and debates about child-rearing among diverse stakeholders, including the bidirectional and dynamic interactions with the digital child-rearing program.

## Methods

### Thrive by Five International Program

#### Overview

The objectives of Minderoo Foundation’s Thrive by Five International Program were to (1) empower parents with the knowledge they need to support the healthy development of their child and (2) ensure universal access to this valuable parenting information regardless of location or demographic background. In accordance with the World Health Organization’s classifications of digital interventions in health, the program is primarily a digital health intervention that “transmits health information to person(s) based on health status or demographics” [41], that is, parents or caregivers of children aged ≤5 years. While the Thrive by Five app was the flagship of the program, it is important to highlight that a multichannel approach to implementation was used to broaden access beyond the app, including other digital (eg, WhatsApp [Meta Platforms, Inc]) and nondigital methods (eg, pamphlets, cards, and workshops) as appropriate for the context. As a universal intervention, the program was designed with the intention of being appropriate and relevant to all parents and caregivers of children aged between 0 and 5 years [42].

The Youth Mental Health and Technology Team from the University of Sydney’s (Australia) Brain and Mind Centre was the research and evaluation partner for this large-scale global initiative. The program (described in greater detail in subsequent sections), including associated content, app features and functions, implementation plans, promotion and dissemination strategies, and evaluation methodologies, was iteratively informed through learnings from research conducted from 2021 to 2024 across 10 LMICs in Africa (Cameroon, the Democratic Republic of the Congo [DRC], Kenya, and Namibia); Central Asia (Afghanistan, Kyrgyzstan, and Uzbekistan); Melanesia (Papua New Guinea [PNG]); and Southeast Asia (Indonesia and Malaysia). Importantly, the first 2 countries (ie, Afghanistan and Indonesia) were the proof of concept for the Thrive by Five International Program. The research team’s approach to developing, testing, refining, and evaluating the program aligned with the MRC framework, including the use of mixed quantitative, qualitative, and process methodologies [43-45]. Across the project, the countries in which the sites were located were grouped by geographic regions including Africa, Central Asia, and Southeast Asia. Countries with larger populations were typically slated for earlier implementations in each region as they afforded more opportunity for program learnings. These learnings were then fed forward and translated into R&D processes for other sites in the same region. It is important to note that the evaluation did not proceed in PNG and the DRC due to issues with feasibility, as most evaluation activities were conducted using web-based programs (ie, REDCap [Research Electronic Data Capture; Vanderbilt University] and Zoom [Zoom Communications, Inc]). In addition, only the process evaluation interviews were conducted in Kenya.

#### Program Content

**Figure 1:**
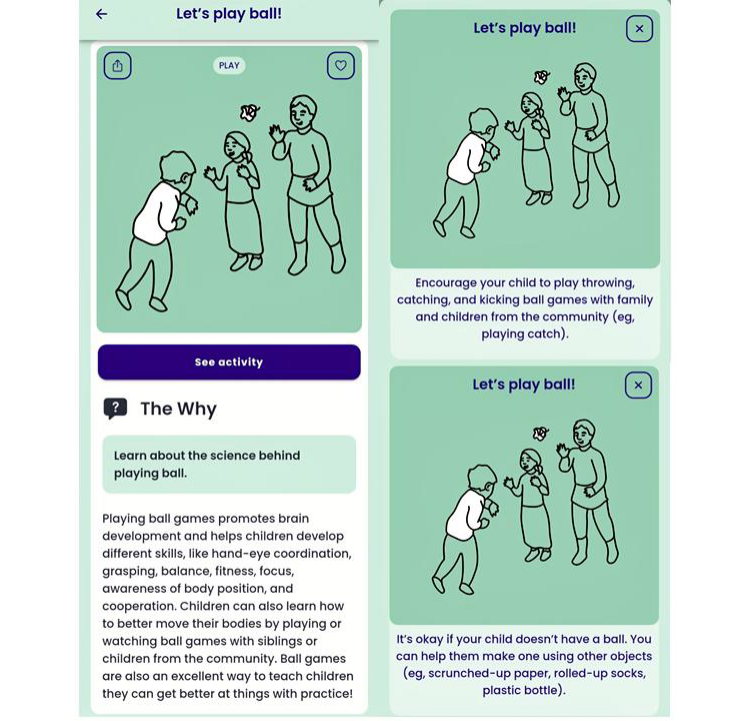
Example collective action.

Referred to as collective actions, the content provides parents and other caregivers with evidence-based information about early childhood development coupled with practical activities that can easily be carried out in the real world with commonplace household or natural materials (eg, alternatives for toy balls included scrunched-up paper, rolled-up socks, and plastic bottles; Figure 1). For each country, the content was localized through the inclusion of examples of local children’s stories, songs, dances, games, locations, foods, customs, and traditions. Importantly, all local examples were reviewed and approved by parents and subject matter experts for familiarity and appropriateness. The library of content developed over the course of the 3-year project, with new collective actions being developed for most countries through the co-design process. Participants from all countries agreed that existing content was relevant to parents and families in their country, with the exception of Afghanistan, where any activities that referenced singing that might be done outside the home were removed owing to public restrictions on such activities. Recognizing the benefits for children of engaging with multiple caregivers in early childhood, the content encourages the involvement of parents, including specific reference to both mothers and fathers, as well as grandparents, aunts and uncles, older siblings, and trusted members of the community.

#### Implementation and Promotion

The finalized content was distributed primarily via country-specific iterations of the Thrive by Five app available for free through app stores and via other digital (eg, WhatsApp chatbot) and nondigital methods (eg, printed materials). Implementation and promotion were led by Minderoo Foundation in collaboration with the in-country partner, with promotion strategies tailored to the preferences of target end users. This included digital advertisements through social media including the use of influencers, print advertisements on public transport, family days at local parks and shopping malls, television and radio broadcasts, YouTube videos, and emails through professional networks. In some instances, Minderoo Foundation partnered with a professional marketing team (ie, M&C Saatchi and Magenta) to create targeted advertising campaigns to promote the program.

### Multicase Study Design

**Figure 2:**
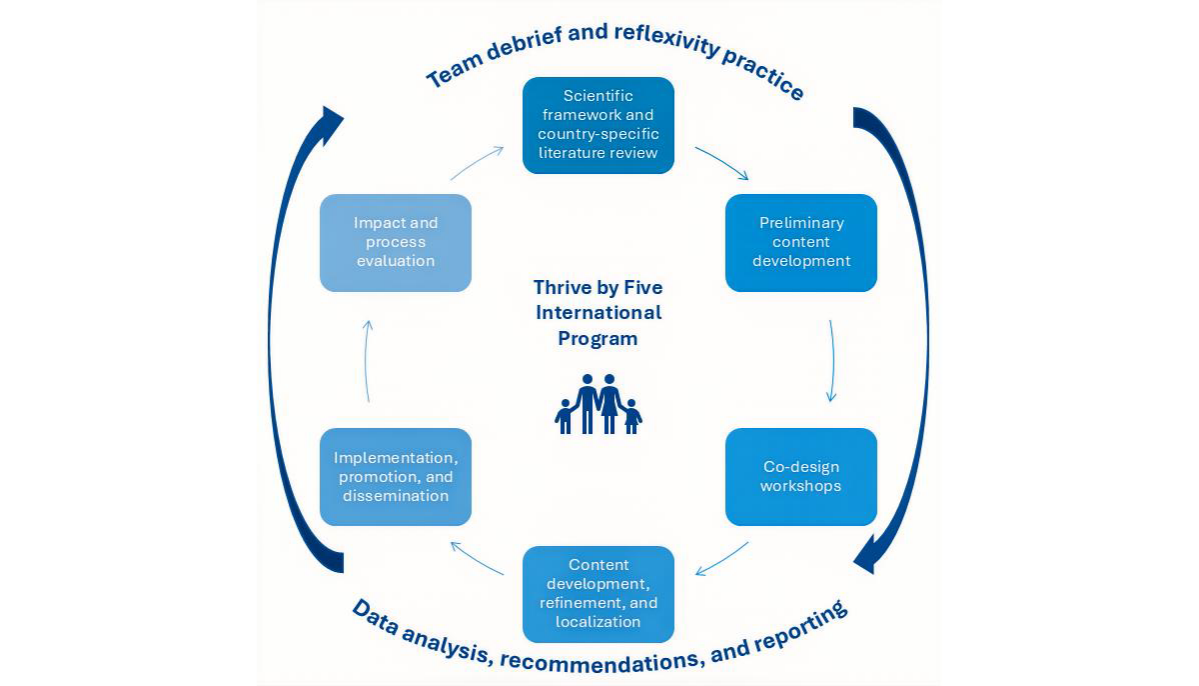
Phased approach to mixed methods data collection and analyses across cases.

This study was influenced by the multicase study design by Stake [46,47] in which multiple cases are analyzed to explore and build understanding of broader research questions. The collected cases are bound together by the program, which Stake [47] refers to as the “quintain” or broad target of study. The individual cases were studied in-depth to develop an understanding of their situational uniqueness, with data analyzed iteratively to examine the dynamic and evolving interactions between program processes and context [38]. Learnings were then synthesized across cases to inform comprehensive understanding of the quintain across diverse contexts [47]. This multicase approach informs a deep understanding of the program as well as a nuanced appreciation for how it is influenced by various sociocultural and contextual conditions [47]. For the purposes of this study, a case was defined by the country in which the research was conducted. As depicted in Figure 2, for each individual case, the research was conducted in phases in accordance with standardized research frameworks and protocols [43-45], with learnings being fed forward into future cases.

### Research Methodologies

All research frameworks and methodologies have been published previously and will only be summarized in Table 1, with further details available in Multimedia Appendix 1 [28,43-45,48-57]. We have also provided a summary of the number of workshops and interviews and corresponding sample size for the co-design, impact, and process evaluation research in Table 1.

**Table 1:** Research methodologies and number of participants.

Research method	Description
Scientific framework [43]	The program is underpinned by a scientific framework highlighting key neurobiological systems that are involved in social, emotional, and cognitive development, including the stress response system, the oxytocin system, the learning system, the fear-arousal-memory system, and the circadian system.
Literature review [43,44]	For each case country, the research team conducted a comprehensive literature review to build understanding of common child-rearing practices and behaviors, family dynamics and relationships, personal and societal values, and broader sociocultural and contextual factors that may influence family and community functioning and, in turn, early childhood development. The learnings from this review informed the preliminary content development.
Co-design methods [44]	After testing the preliminary content, parents, caregivers, and subject matter experts participated in a series of co-design workshops to actively contribute to the development and refinement of the content, informing its cultural appropriateness, contextual relevance, localization, and alignment with user needs. A sample workshop agenda has been provided in Multimedia Appendix 2. Across the 10 LMICs^a^, 41 co-design workshops were conducted with 216 parents and other child caregivers. In addition, 18 co-design workshops were conducted with 70 subject matter experts. Alam et al [58] provides a more comprehensive report of participant demographics.
Impact and process evaluation methods [45]	After a 2- to 24-week period of engaging with the program, parents and child caregivers provided their feedback on the impact and effectiveness of the program through qualitative semistructured interviews and workshops. Quantitative surveys were also conducted; however, only qualitative feedback about the translation of and experiences completing these surveys is included in this study. No specifications were set for evaluation participants with regards to frequency of app and content use, time spent on the app and content, level of app and content engagement, or number of collective actions completed. The primary objective of this mixed methods study was to evaluate the impact of the program on parent and caregiver knowledge, behaviors, attitudes, and confidence, as well as the connection between the child and their parents, family, community, and culture. A total of 29 impact evaluation interviews and 12 workshops were conducted with 86 parents and other caregivers. Loblay et al [30] provides a more comprehensive summary of participant demographics. Key collaborators and stakeholders, such as staff members from Minderoo Foundation and in-country partners, actively involved in R&D^b^ processes were also invited to participate in opportunistic semistructured and conversational interviews. These interviews were designed to explore: the system change processes supporting and shaped by the program, how partnerships were influenced by sociocultural and contextual factors, and challenges experienced by collaborators when establishing partnerships and implementing the program. Schedules for the impact evaluation interviews and workshops, as well as the process evaluation interviews, have been provided in Multimedia Appendices 3-5. In total, 18 process interviews were conducted with 8 in-country parents or subject matter experts and 10 research collaborators. The overarching results of the impact and process evaluations have not been published; however, qualitative data from Indonesia specifically highlighted the importance of social connection and interactive community-based and multigenerational learning to enhance engagement with and, in turn, impact of the program [30]. Furthermore, quantitative survey data from parents in Indonesia, Malaysia, Afghanistan, Kyrgyzstan, and Uzbekistan highlighted that better emotional adjustment is associated with more positive parenting practices, suggesting that interventions that support a parent’s ability to cope with the parenting role will increase the use of responsive and nurturing child-rearing practices [59].

^a^LMIC: low- and middle-income country.

^b^R&D: research and development.

#### Recruitment and Data Collection

Recruitment for all research activities was led by the site principal investigator from the in-country partner in each respective country using their established networks and advertising mechanisms. The respective sites used the recruitment methods best suited to their community and context (eg, emails, poster displays, paper-based and online internal news articles, handouts, and digital advertisements on social media). The research team worked directly with the sites to discuss how best to recruit a diverse sample of participants as well as to manage any cultural and contextual challenges as they arose. Sampling methods differed based on the networks and reach of the respective in-country partners, with varying impacts on R&D processes, which is discussed in detail in the Results section as one of the learnings from this multicase study. Co-design participants were recruited from 10 countries, with purposive sampling methods used in Indonesia, Kenya, Malaysia, and Namibia and convenience sampling used in all remaining countries (ie, Afghanistan, Cameroon, the DRC, Kyrgyzstan, PNG, and Uzbekistan). Impact evaluation participants were recruited from 7 countries. Convenience sampling methods were used for recruitment in Afghanistan, Cameroon, and Namibia whereas purposive sampling strategies were used in Indonesia, Kyrgyzstan, Malaysia, and Uzbekistan. Process evaluation participants were recruited based on their degree of knowledge with regard to the factors that impacted R&D processes.

All interviews and workshops were conducted remotely via Zoom, a secure cloud videoconference service with end-to-end chat encryption, with a translator available as needed. Translators for co-design workshops were provided by the in-country partner, whereas National Accreditation Authority for Translators and Interpreters accredited translators were used for all evaluation research activities. National Accreditation Authority for Translators and Interpreters is the national standards and certifying authority for translators and interpreters in Australia [60]. With participant consent, the research activities were audio and video recorded and transcribed verbatim to ensure all discussions were captured accurately. In addition, 1 to 2 scribes from the research team were present to take written notes. Finally, quantitative survey data were collected via REDCap, a secure electronic data collection and management tool [48,61] hosted by the University of Sydney.

### Data Analysis

The data analysis for this study occurred across 3 iterative phases (Multimedia Appendix 1 provides more detail about the phase 1 and 2 analyses). First, interpretation of the qualitative data from the semistructured interviews and workshops followed established thematic techniques [49]. All raw data were reviewed and checked across all participants by senior researchers to develop a coding framework outlining all key concepts. Subsequently, data were coded in NVivo (version 14; Lumivero) software [62] using this framework, adhering to an established iterative process of reading, coding, and exploring the pattern and content of coded data, followed by reflection and discussion to reach consensus. The key lessons learned from the co-design and evaluation research for each case were synthesized in outcome reports.

Second, at the conclusion of each case, an overarching transfer of a learnings report was produced to integrate the learnings from across all research activities to inform future iterations of the collective actions and features and functions of the Thrive by Five app, both for the current case country as well as for other cases that had not commenced or were at earlier phases in the R&D cycle. In addition, successes and challenges relating to R&D processes, implementation, promotion, and dissemination were summarized, including suggestions for how to champion or mitigate these factors. The details of these reports varied; however, an indicative sample of topics is highlighted in Textbox 1. Formal recommendations were documented to iteratively inform R&D processes as well as program features and content.

**Sample topics summarized in transfer of learning reports.**

- App features, functions, and content that required review and refinement.
- Demographic and sociocultural factors that influenced engagement with and adoption of the program, including how this might shift based on the dynamic relationships between different caregivers (eg, mother and father and mother and mother-in-law).
- Effective or recommended methods of promoting and disseminating the program at a community level, such as through community leaders or family elders.
- Suggested messaging to promote interest in and engagement with the content (eg, to promote children’s school readiness).
- Unexpected outcomes in relation to parenting behaviors, practices, attitudes, and confidence.
- Process-level concerns raised by collaborators relating to language and translation, promotion and dissemination strategies, partnerships, and timelines.

Third, at the conclusion of all individual case data collection, primary analysis, and reporting, the research team met to nominate, discuss, and synthesize learnings from each case in accordance with the MRC framework. Informed by the approach to multicase studies by Stake [47], the program was used to bind the learnings across diverse contexts. Following discussion, these learnings were integrated into a basic framework for the results by HML. To further expand on these learnings, senior researchers (HML and VL) subsequently conducted a joint secondary analysis of the research data, focusing on concepts relating to program theory, stakeholder engagement, and the iterative refinement of the program as they related to, interacted with, and were influenced by the sociocultural context.

### Team Debriefs and Collective Reflexivity

Throughout the research activities for each case, the research team engaged in routine debrief sessions to reflect on research processes and learnings. This provided an opportunity to engage in collective reflexivity as a team. Reflexivity is “the extent to which group members overtly reflect upon and communicate about the group’s objectives, strategies (eg, decision-making) and processes (eg, communication), and adapt them to current or anticipated circumstances” [63]. In multidisciplinary research, reflexivity is a tool used to improve communication, foster a shared vision of the research objectives and methods, build understanding from the diverse perspectives and skills of members of the research team, and develop innovative strategies to resolve conflicts and overcome research challenges [64]. In this setting, the research team was comprised of researchers with not only diverse disciplinary training but also a broad range of cultural and ethnic backgrounds that traversed WEIRD countries (Australia, England, and the United States) and LMICs (Bangladesh, Iran, India, and Pakistan) as well as Indigenous backgrounds. Reflexivity practice enabled researchers to reflect together on the cultural particularities of their own child-rearing experiences in relation to the research learnings, reinforcing awareness of the plurality of approaches to child-rearing. These discussions also iteratively informed decision-making regarding mixed methods research approaches and facilitated exploration of differing perspectives of research team members and how these influenced understandings of context [65].

### Ethical Considerations

This study was approved by the University of Sydney Human Research Ethics Committee (HREC; project 2021/956); the Committee on Bioethics at the Global Research Institute Foundation, Kyrgyzstan; the University of Respati Indonesia; the SEGi Research Ethics Committee, SEGi University, Malaysia; the Ministry of Social Affairs, Republic of Cameroon; the National Health Ethic Committee for the Ministry of Public Health, DRC; the University of Nairobi Faculty of Health Sciences and Kenyatta National Hospital, Kenya; and the National Commission on Research, Science and Technology, Namibia. At the time that this research was conducted, there was no local ethics committee operating or established social science and ethics standards in Afghanistan, Uzbekistan, or PNG. Therefore, a site-specific protocol and supporting documents were approved by the University of Sydney HREC. For each site, the site principal investigator assisted in identifying and providing advice on the appropriate country-specific HREC as well as with the preparation and submission of an ethics application as required. In accordance with ethics approval, participant information statements were available for and specific to each site. All participants were adults aged ≥18 years and provided informed consent before engaging in the research activities. Qualitative data collected via interviews and workshops were deidentified at the point of transcription to protect the privacy of participants and stored separately from consent records. Incentives to compensate participants for their time were offered based on each site’s national paid participation rates and methods of reimbursement as recommended by the local ethics practices and committee’s advice.

## Results

Guided by the core elements of the MRC framework, the data were synthesized into five themes: (1) the role and value of partnerships, with an emphasis on partnerships with in-country organizations and governments; (2) building collaborative practice with partners and stakeholders; (3) honing a target audience; (4) navigating the digital landscape; and (5) managing linguistic diversity and translation. As described previously, sociocultural context was considered a dynamic process rather than a separate entity to be studied; therefore, learnings regarding context permeate all results.

### The Role and Value of Partnerships

#### Overview

Importantly, parents, other caregivers, and subject matter experts (eg, clinical psychologists, early childhood educators, medical specialists, anthropologists, and linguists) were key stakeholders throughout the R&D processes, providing invaluable insights to inform the program design and iterative refinement across diverse contexts. Indeed, it was universally agreed by all partners that direct and continual engagement with community members was the cornerstone of this project. The co-design and evaluation learnings were extensive and have been reported elsewhere [28,30,58,59,66]. Therefore, this section focuses on partnerships with in-country partner organizations and governments.

#### In-Country Partners

Before embarking on research in each country, Minderoo Foundation was responsible for scoping, selecting, and establishing all relationships with partnering organizations. Typically, this was initially handled by their partnerships team, following a strict due diligence process, and subsequently led by Minderoo Foundation’s Thrive by Five International Project Team. In-country partners provided on-the-ground support, guidance, and expertise throughout the duration of the project. As shown in Table 2, all partners were nongovernmental organizations except for Uzbekistan, where the partner was a subsidiary of the Ministry of Preschool Education.

**Table 2:** In-country research partners for each case country.

Case	Research partner
Afghanistan	Bayat Foundation
Kyrgyzstan	Roza Otunbayeva Initiative
Uzbekistan	The Innovation Centre for Information and Pedagogical Technologies, a subsidiary of the Uzbekistan Ministry of Preschool Education
Indonesia	The Indonesian Breastfeeding Mothers Association, The Indonesian Child Welfare Foundation
Malaysia	Malaysian Association of Professional Early Childhood Educators
Papua New Guinea	World Vision PNG
Cameroon	Kalkaba Development Initiative
Democratic Republic of the Congo	Mission in Health Care and Development
Kenya	Shining Hope for Communities
Namibia	Development Workshop Namibia

All partners were enthusiastic and passionate about promoting positive child-rearing practices and improving outcomes for children and their families. However, the established networks for each partner varied considerably in terms of diversity and reach, thus impacting R&D processes, including recruitment strategies. For example, the Indonesian Breastfeeding Mothers Association promotes breastfeeding primarily through web-based communication and digital media. This organization was responsible for recruiting participants to the co-design workshops as well as the promotion and dissemination of the app. Their network enabled recruitment of participants from diverse backgrounds and with varying levels of education and socioeconomic status. However, the marked absence of male caregivers in the co-design workshops and in subsequent research activities suggested the need to broaden the partnership base to include organizations better able to target male caregivers. As another example, the Malaysian Association of Professional Early Childhood Educators (MAPECE) is a nonprofit early childhood care and educational organization whose members are primarily preschool teachers. Owing to their links to preschools throughout the country as well as SEGi University’s early childhood education program, the team from MAPECE was also able to recruit a diverse sample of parents and caregivers for research activities. However, as the professional network of early childhood educators was relatively narrow, the organization was unable to reach the broader population base of parents and caregivers at the point of program implementation.

Importantly, variability in partners’ experiences with and knowledge of research may provide a valuable opportunity for capacity building in relation to sampling, recruitment procedures, and informed consent. Over the course of this project, the research team provided a standardized training for site principal investigators and any interested members of the in-country partner research teams with regard to the evaluation research protocol and consenting process. The training was designed to ensure that they understood and could explain to participants the study aims, eligibility criteria, research activities, including expected duration of participation, and any potential risks or benefits to participation. Furthermore, given the homogeneity identified in early research samples, the research team took a more active approach to working with in-country partners to strategize how to improve representativeness within the sample and how these potential participants might be recruited.

Notably, the research team also benefitted markedly from working with diverse partner organizations, expanding their own skill sets with regard to conducting research in LMICs. This included active practice of cultural humility [67] as well as greater understanding of the intricacies of language translation and interpretation and the need for flexible research methods to promote inclusivity in research activities. As an example, results of the quantitative evaluation survey from Malaysia showed substantial differences across language groups (ie, Malay, Chinese, and Tamil) in relation to app usability as well as cultural appropriateness. However, collaborators from MAPECE cautioned the researchers to avoid equating language of survey completion with ethnicity, noting that these findings were only attributable to survey respondents and should not be generalized beyond this group.

#### Government Stakeholders

Importantly, this section has been anonymized given the ongoing working relationships with governments. Governments played variable roles in each country, ranging from no involvement to providing program approval, promotion, and dissemination. Regardless of whether a government was the identified in-country partner, it was often “crucial to have government buy in from the beginning” (Minderoo Foundation collaborator, evaluation interview). Across cases, this was typically most impactful at the point of implementation, including specific promotion and dissemination activities, as stressed by an in-country collaborator who noted the “need for authorisation from the authority to do something like this [webinar] such as the [governmental body]. So they would provide a letter of encouragement to participate.” In this instance, there was minimal government buy-in when the program was implemented in the country and, therefore, no support for dissemination activities.

In another case, it was not possible to engage government officials in discussions about the program despite multiple attempts, and the R&D processes proceeded without their involvement. Just before implementation, government representatives provided details as to the multistep requirements for the review, validation, and implementation of programs such as Thrive by Five. Despite a positive response to the program content and praise for the community-based research methodologies used in its development, it became clear that there were several steps in place that would be required to progress toward implementation.

In a third case, the government endorsed the program and promoted it via their social media channels, but direct dissemination through the government was not possible due to organizational changes.

Finally, in another instance, Minderoo Foundation actively involved the government in the design, development, implementation, and promotion of Thrive by Five, including providing specific input on the appropriateness of the content for the local context. Notably, the government sought to broaden the scope of the project, including additional translations of content and evaluation materials. The government also sought to lead the evaluation but was ultimately satisfied by the explanation that the research was designed to be conducted by the University of Sydney research team independent of both Minderoo Foundation and the government. Importantly, the research team explained that outcome reports, including aggregate data, would be available for review via the in-country partner. The in-country partner was critical in managing and stewarding the relationship with the government throughout these discussions.

### Building Collaborative Practice With Partners

Minderoo Foundation’s statement of need for the program (unpublished) read as follows: “All parents and caregivers need support and guidance in lifting the cognitive, socioemotional and brain development of their children under 5 years of age.” Aligned with this, the program vision was threefold: (1) empower parents with the knowledge they need to support the healthy development of their child; (2) ensure universal access to this valuable parenting information regardless of region, socioeconomic status, literacy, gender, or other barriers; and (3) develop strong partnerships with in-country organizations to validate the cultural appropriateness and relevance of the app content as well as its features and functionality.

While the vision for Thrive by Five may have been documented, the in-country partners from Indonesia, the first proof-of-concept case, found it difficult to join in a project where this vision had not been established collaboratively:

When we were developing the whole thing, we were not starting from the same starting point, we did not know the vision so we could not work towards that vision. But when we finished, we finally realised what the vision was, but we had to get through a lot to get to that point.

Indonesia collaborator, evaluation interview

Furthermore, others cited a missing “sense of ownership” from the partnership dynamics:

I would have liked the process to start with a discussion, say let’s have a discussion we want to develop this program, and then we would be able to offer suggestions about the material and medium. In terms of the process itself it would be a good idea to explore it together rather than being given the material and then being asked for the input.

Indonesia collaborator, evaluation interview

More generally, there was clear acknowledgment from both Minderoo Foundation and Indonesia collaborators that the agreed-upon timelines for the proof-of-concept case were too tight (“it’s very intensive, it’s very full on” [Minderoo Foundation collaborator, evaluation interview]), creating a time burden that considerably exceeded expectations. Therefore, at times partnership interactions were perceived as “very transactional” (Indonesia collaborator, evaluation interview) as there was a lack of appreciation of the pace and rhythm of work in the Indonesian context.

Importantly, these learnings were fed back to Minderoo Foundation through the evaluation reporting and underscored the need to more explicitly discuss how to work best together and maintain clear lines of communication with future partners throughout the project life cycle and beyond:

There’s been lots of learning since, about how we can do things better and the partnerships... since then, we’ve actually hired a partnerships manager, because we realised that we really needed someone to be able to be there from the first acquisition period through the whole lifecycle.

Minderoo Foundation collaborator, evaluation interview

These learnings resulted in marked changes in the collaborative nature of partnerships between Minderoo Foundation and in-country partners as the project progressed, as exemplified by this collaborator:

So I think the partnership has worked very well 90% of the time, with Innovation Centre and Minderoo. It seems like we had a common ground, we had a common sense and understanding of what is needed.

Uzbekistan collaborator, evaluation interview

Minderoo Foundation also decided to co-design all aspects of the work plan guiding the collaboration with the in-country partner to ensure a shared vision, a collaborative approach to working together, and feasible expectations with regard to timelines and deliverables.

Notably, in addition to establishing a collaborative working relationship with in-country partners, it was also critical to build trust with potential end users about the motivations for the program. This is best exemplified by the wariness expressed by a mother from Kyrgyzstan:

Western methods, of course, are not perfect. Maybe we are authoritarian or strict but in Western countries they allow everything. There is too much freedom for the children. I think this is not fully correct. Children should understand where the truth is and what is wrong. In our mentality, there is a lot of positive in our upbringing. We are taught to respect elder people. In the West, they grow up with a big ego... they are not taught to respect elder people.

Kyrgyzstan parent, co-design workshop

A father from Malaysia echoed this, saying:

Western and Eastern ways of thinking and teaching children, there are some differences. Here in Chinese culture, we give respect, we respect others, we give way to people. It is a good habit or attitude over here.

Malaysia parent, co-design workshop

Indeed, this comment highlights the perceived distance between researchers and participants. Importantly, the research frameworks and protocols were specifically developed to ensure local cultures, customs, and values were reflected in the program [43,44]. Nevertheless, experiences such as these emphasized the need for each research team member to acknowledge their own assumptions and values with regard to early childhood development and to recognize the impacts that they may have on those participating in the research. It was critical to recognize the inherent power dynamics, particularly in relation to real or perceived differences in the attitudes, beliefs, and values of researchers and participants from the Global North and Global South, to work toward equitable collaboration. As part of this, it was important to explicitly emphasize to research participants that the program could not be successful without their cultural and contextual expertise (ie, “We are here to learn from you” [research team member, co-design workshop]). In addition, it was always helpful if the cofacilitator or other representative from the in-country partner reassured participants about speaking openly and honestly, “I am telling them we want them to be frank, to talk easily and freely” (DRC collaborator, co-design workshop). While it is naive to suggest that such statements eliminated power imbalances, they helped to position research participants as highly valued contributors to R&D processes.

### Honing the Target Audience

Thrive by Five was always intended as a universal child-rearing program to empower parents and caregivers with evidence-based information about early childhood development and practical activities they can engage in with their young children to support socioemotional and cognitive development. However, we consistently found stakeholders were “confused whether the app is for parents or for children” (Indonesia expert, co-design workshop), particularly in the earlier cases. In Afghanistan, a parent stated the following:

I think the kids would find this boring and then they don’t want to connect with their parents. If there is something we can have to attract kids, then we may have a better connection with them.

Afghanistan parent, co-design workshop

Across all cases, there was also considerable discussion about how best to support parents and caregivers with children with disability through the content. For example, we heard from experts, “My first observation is that it could perhaps be more inclusive of children with disability...it should include more content for children with disability” (Namibia expert, co-design workshop), and “They don’t go into consideration of children with slow development” (Cameroon expert, co-design workshop). Parents also wanted more information about how best to track child development milestones and skill development to enable clinical diagnosis.

In response to the ongoing confusion from stakeholders with regard to target end users and intervention type (ie, universal), the research team developed the explanatory disclaimer presented in Textbox 2 to help clarify the intended audience and program scope before commencing the R&D process. Additionally, as we had been told the following:

Most of the children with disabilities are kept away. Letting the parents know it is not a shame…...educating them that it is not the end if the child has disabilities, there is more they can do for this child. ([Namibia expert, co-design workshop])

We took this opportunity to actively highlight the potential applicability of the collective actions for children with disability with accommodations. However, content specific to children with disability was outside the scope of the program. The intention was for this type of information to be included as part of all content dissemination strategies.

**Explanation of the intended target audience for Thrive by Five.**

- Thrive by Five is an app for parents, guardians, and other caregivers. The purpose of Thrive by Five is to provide parents and caregivers with evidence-based and culturally appropriate information about early childhood development, along with tips for parenting practices. Along with parents, the activities encourage the involvement of the child’s wider family, such as grandparents, cousins, aunts, and uncles.
- Young children are sensitive to external influences that shape their development, and all children benefit from an interactive and caring environment irrespective of their age, developmental stage, or ability. Like all children, children with disability thrive in settings with plenty of learning opportunities and bring their own strengths to interactions with others. This app does not provide content specific to children with disability; however, it encourages parents and the wider family to interact, play with, and teach children (including children with disability) at home and in the community, and outdoors when possible. Each collective action includes suggested activities. Some children, including children with disability or impairments, may find it difficult to do some of the activities. We encourage doing activities within the child’s capability. Children vary in their developmental progress and in reaching milestones; if there are concerns about a child’s development, the Thrive by Five app cannot replace clinical or professional assessment and intervention.

Despite the conceptualization of the program as a universal intervention, stakeholders frequently expressed that it is impossible to reach the whole population within a country, stating, “to develop something that is supposed to target one group of individuals, another group may be left out” (Kenya expert, co-design workshop). Challenges were noted regarding regionality, varying levels of education, languages spoken, and internet and smartphone access within the country; therefore, it was recommended that a well-defined dissemination area and target audience were required to facilitate program success. It was also recognized that defining a specific target group and/or region would also provide greater clarity to the in-country partner as to who should be recruited to participate in research activities:

It is extremely difficult, almost impossible, or not possible to embrace the whole population of the country because the levels are very different. So if you would like the people in metropolitan areas, people living in the cities, in towns to use this application, that should take into account just a number of factors. But if you want people living in the country to be the users of your application—and that’s the majority because really the majority lives in the country, Internet access itself is a challenge. Also, you need to take into account the educational level of people living in the country, their level of education is much lower than the level of education of people living in metropolitan areas.

Uzbekistan collaborator, evaluation interview

With this in mind, Minderoo Foundation developed three personas that more clearly defined the target cohorts for the program: (1) parents and caregivers with low levels of education and income who likely live outside of urban areas, (2) more educated parents and caregivers with low- to middle-income levels who likely live in urban areas, and (3) educated community leaders with a strong circle of influence on personas 1 and 2. Notably, the importance of targeting community leaders was in part a product of evaluation learnings that highlighted the importance of bottom-up promotion and dissemination.

### Navigating the Digital Landscape

The Thrive by Five app was originally envisioned as the primary means of content dissemination for the program; however, concerns regarding the availability of digital technologies as well as electricity and internet access were noted to impact R&D processes across all cases. Specifically, unreliable internet connections frequently resulted in disruptions during routine project planning and management meetings with in-country partners, as well as co-design and evaluation activities with stakeholders. Due to budgetary limitations and safety concerns related to conducting field research in some countries, the sample of participants available for research activities was restricted to those who could access electricity, the internet, and a smart device or computer, which often resulted in unrepresentative samples comprised primarily of individuals who lived in urban settings and who were more highly educated. This was reflected in participant demographic data collected by the in-country partner or via the research team’s evaluation survey.

These infrastructural issues were anticipated to be a barrier for parents and caregivers being able to access the program. Experts explained as follows:

One of my concerns, I don’t think we are reaching the majority of parents by only being online—that is just a general concern from what we are seeing with our programs.

Namibia expert, co-design workshop

It can be challenging because many rural areas may not have access to internet and smartphones...the same applies in urban informal settlements where parents are struggling to feed their children.

Kenya expert, co-design workshop

Such distinctions in internet and device accessibility in rural compared to urban settings were common, as highlighted by a Malaysia expert: “we need to be very careful about which segment of society that you want to target because...with the poorer community where we talk about accessibility of internet you know, in rural areas they cannot access it so how can they go into the app” (Malaysia expert, co-design workshop). An expert from PNG noted that a previous digital education project had to provide solar panels and tablets as the schools did not otherwise have access to electricity. Indeed, download numbers as of January 2024 reflected infrastructural issues, with the fewest downloads in the DRC, PNG, and Cameroon. Notably, the in-country partner in Afghanistan helped overcome these infrastructural barriers. The Bayat Foundation is a member of the Bayat Group, Afghanistan’s largest private diversified service company, which includes telecommunication companies (ie, Afghan Wireless, Ariana Television Network, and Ariana Radio Network) that enabled the successful implementation and reach of the program despite the active conflict in the country.

Recognizing barriers to app access, Minderoo Foundation worked with in-country partners to identify alternative means of dissemination, that is, a multichannel approach. For example, in Indonesia, *socialization* workshops were conducted with parents and caregivers to share the content in a group-based learning environment. These were noted to be particularly impactful in rural communities, where “the enthusiasm was clearly different. Mothers came very enthusiastic, came from 8am when we start at 9am...participants taking it as their knowledge to bring it home and ready to share with others” (Minderoo Foundation collaborator, evaluation interview).

As detailed in the paper by Loblay et al [30], identifying and empowering community leaders and local change agents in Indonesia proved to be another critical means of bottom-up program dissemination. In Namibia, paper booklets of the content were created, and “there were also these beautiful radio plays that kind of amalgamated our content” (Minderoo Foundation collaborator, evaluation interview). Importantly, as the program was found to be “doing better in those more underserved settings,” Minderoo Foundation increasingly placed greater emphasis on “needs based level of support...this determines exactly where we go and then we will try to support and target” (Minderoo Foundation collaborator, evaluation interview), informing the development of multichannel dissemination strategies that did not rely on the Thrive by Five app.

Beyond issues of accessibility, the marketplace for digital child-rearing programs such as Thrive by Five was highly variable across cases. For example, we heard from a parent in Kyrgyzstan that “we have almost no parenting applications available for aged 0-5” (Kyrgyzstan parent, co-design workshop). Conversely, our review of available academic and gray literature found that parenting apps are popular in Malaysia, including some that had the potential to compete with Thrive by Five in relation to targeting socioemotional development (eg, Asianparent: Pregnancy and Baby) [68]. In addition, Facebook, Instagram, and TikTok were frequently cited in co-design workshops as social media resources parents, particularly new mothers, accessed to learn about development and child-rearing. Despite concerns raised by experts regarding the accuracy of the information being conveyed, the brief and engaging nature of these social media platforms was a source of competition for the more comprehensive and evidence-based Thrive by Five app, as reflected by a mother from Indonesia who stated:

Probably an Instagram feed reel that we can easily access in a short chunk of information, and in real time because [on] Instagram, every day, there is new content to watch, and the content is given verbally in a short video.

Indonesia parent, evaluation interview

### Managing Linguistic Diversity and Translation

Feedback on the content translations was common. For example, in Indonesia, we heard, “I think the translation is too literal and not localised...too rigid. Sometimes I find it very difficult to understand the text...we need to localise it to make it more understandable” (Indonesia expert, co-design workshop).

Furthermore, in this case, translation of the content extended the length of the content, as more words are required in Bahasa Indonesian when translated from English. It was noted that this may be overcome if the content was written in the local vernacular as opposed to being more of a direct translation from English. The need to tailor to the context was reiterated in other cases:

When we do the translations there are words we need to work around, and then it will probably give the proper context. It will work, but we will need time to write it out plain and simple.

PNG expert, co-design workshop

As the critical importance of translation came to be appreciated during the initial cases, Minderoo Foundation allocated more time for the in-country partner to facilitate high-quality translations. This increased time allowed the translations to be approached with considerable care and enabled iterative refinement based on expert feedback. In Uzbekistan, the in-country partner highly valued the patience and understanding in relation to iteratively revising the translation of the content and for the cultural competence of the program:

We had to localise, adapt the content over and over again, to make sure that we do the language easier, so that people will be exposed to it and understand it easier. So they were very patient, they were understanding. It is something very, very rare in partners, because once things set, some of the partners do not really want to change anything, but it is good that we do have that understanding and we do believe that the app should succeed. So for the app to succeed, we have to make some changes. And that was great.

Uzbekistan collaborator, evaluation interview

The Kyrgyzstan case highlighted the value of including a translator as a member of the in-country partner team from the beginning. Here, the primary translator attended project management meetings to develop a strong understanding of the objectives of the program and participated in co-design workshops to learn about the unique needs of parents in this context. When subsequently translating the program content, this translator helped refine terminology to ensure the meaning of the content was preserved but also understandable to users. For example, “patterns” is used in a collective action in relation to mathematical principles. The translator noted that in Kyrgyzstan “patterns” would only relate to fabrics; therefore, this word was changed to “similarities.” The translator’s advice also extended to the approach to phrasing to ensure that the tone of the collective actions reflected ways that people from Kyrgyzstan are used to learning. It was emphasized that the content should be directive rather than encouraging (ie, “Try to find ways to show your child...” was changed to read “Show your child...”).

Challenges related to language extended to the quantitative evaluation surveys. This appeared to relate less to the translations of the language and more so to the familiarity or comfort with the types of questions in the survey, particularly in relation to mental health and well-being. For example, feedback conveyed by our collaborator in Uzbekistan included “How do you rate happiness?” Similarly, our collaborator in Cameroon questioned whether participants had adequate familiarity with questions about their mental health to feel confident in providing a response. This appeared to result in a higher frequency of neutral (ie, no change) responses, particularly among male caregivers. These comments underscore the importance of using questionnaires that are reliable and valid when used with the target audience. As no single questionnaire relevant to our identified research objectives had been validated across all contexts, more flexible, context-specific approaches to assessing the same outcomes may have yielded more reliable, high-quality results.

## Discussion

### Key Lessons Learned

This multicase study synthesizes the learnings from an international, multisite program of research using mixed methods to develop, test, and evaluate a digital and nondigital child-rearing program across 10 LMICs. The data synthesis identified five key areas of learning: (1) the role and value of partnerships, (2) building collaborative practice with partners, (3) honing a target audience, (4) navigating the digital landscape, and (5) managing linguistic diversity and translation. Importantly, the dynamic and multifaceted influence of culture and context was integrated across all learnings.

### Fostering Equitable Collaborations

Cross-sectoral partnerships between diverse groups, including nongovernmental and governmental organizations, are increasingly viewed as an effective strategy to address large-scale social and public health challenges, including improvements in service provision and increased public satisfaction [69-72]. Such partnerships typically bring together individuals and organizations with varied expertise and experiences, perspectives, societal positions, and methods of working [73], which ultimately underpin the potential for both success and failure. In this project, there was no doubt that all contributors added value to the project and that partnerships included individuals or groups with the authority to make key decisions to ongoingly progress the work, both of which have been shown to be critical to the feasibility of partnerships [74]. However, the research team found that tight timelines, particularly in early cases, had the potential to undermine the sense of cooperation between Minderoo Foundation and partnering organizations.

The Rethinking Research Collaborative has developed 8 principles to promote fair and equitable research partnerships in the global context, which emphasize recognizing the dynamic, multistakeholder, context-dependent nature of partnerships; accepting uncertainty, ambiguity, and contradiction; and adapting through routine and collective critical reflection for conflict resolution [75]. So-called “productive tensions,” including those between different types of organizations and across disciplines, may be difficult to address but offer an opportunity to actively and collaboratively explore issues related to power, knowledge, and styles of working to build trust and avoid intractable issues [76-78]. In this way, it becomes possible to move beyond a static and simplistic partnership and rather forge equitable collaborations to drive positive change processes [75].

Among the partnership-based variables influencing the success of change projects (eg, communication and readiness for change), a shared vision has been shown to have the highest degree of impact [79]. Furthermore, a shared belief in the importance of change is critical to facilitate the successful implementation of change projects [80]. To arrive at a shared vision, it is essential to involve a diverse group of key stakeholders, beginning from the initial planning stages [81]. In this project, Minderoo Foundation’s vision and goals of the program evolved and were more firmly established during the early proof-of-concept work, meaning uncertainty was experienced by some partners. Following this formative work, later partners benefited from more time in the work plan dedicated to co-orienting toward objectives for change and establishing a common language, methods, and expectations for effective communication. Nevertheless, opportunities may have been missed to harness “friction” to generate ideas for innovation as the program was adapted to new contexts [78,82,83] as suggested by the Rethinking Research Collaborative [75].

The literature highlights that relationships with government bodies occur on a spectrum, ranging from “incidental overlap” (ie, working toward the same goal in parallel) to “collaboration” (ie, a formal partnership is established based on shared goals and strategies) [74]. Notably, nongovernmental organizations, including those in LMICs, are often established due to the failings of the government to address public challenges, thus setting the stage for potential tension, mistrust, and conflictual power dynamics [84]. Apart from Uzbekistan, governments were not viewed as formal partners in this project. Nevertheless, they often had a marked influence on program success, particularly in relation to the management and oversight of implementation and dissemination strategies. Previous qualitative research conducted in Israel found relationships between the government and philanthropic organizations often leverage their respective strengths; that is, philanthropic organizations bring funding to deliver critical new programs such as Thrive by Five, whereas governments enable recognition and promotion [85]. However, a lack of transparent and standardized processes to guide engagements with governments can make collaborations difficult to navigate, as was evident in research investigating relationships between civil society organizations and governments in 4 African countries [86]. Therefore, formalizing the nature of the partnership, defining roles, responsibilities, and expectations, establishing a collaborative approach to working together, and working toward a shared balance of power are likely most effective in establishing a value-adding relationship [85].

### The Clash With Infrastructure

When initiating digital health initiatives, it is critical to consider project feasibility based on the broader digital ecosystem. The *Digital Health Platform Handbook* advises that a context analysis is a necessary precursor to any digital health initiative [87]. This includes assessment of currently available digital health systems, apps, and programs and the ICT infrastructure, underlying infrastructure (eg, electricity grid), and policy relevant to digital health programs and apps. This type of digital technology inventory is intended to identify gaps between regional and/or national ICT infrastructure and the mode of delivery and desired outcomes of the digital health program. In addition, it will highlight where the digital marketplace may already be saturated, suggesting new apps or programs do not meet a user need. In this project, poorly developed ICT infrastructure was ultimately found to be prohibitive in some cases, with the DRC and PNG being the most notable examples. While the research team investigated the availability and use of the internet and technology generally as well as digital health technologies specifically for each case, this review was completed after the decision had already been made to implement the program in the country. A formal context analysis would likely have been invaluable in informing go and no-go decisions with regard to moving forward with the development of a country-specific Thrive by Five app. For instance, analysis of PNG’s regulatory environment at the time of the project would have revealed that the country’s fundamental ICT policy intended to bolster ICT infrastructure (eg, the Digital Transformation Policy 2022 [88]) and safeguard ICT user protections relating to right to information, privacy, and data security (eg, National Right to Information Policy 2020-2030 [89]) was in its infancy and lacked substantial implementation, impacting digital access. Importantly, this is not to say that the program could not be tailored to countries with poor ICT infrastructure, but rather that Minderoo Foundation’s nondigital approaches could be prioritized.

The World Health Organization specifically highlights the risk of digital health interventions increasing health inequities between those with and without digital access and literacy; however, in these cases the digital mode of delivery was not aligned with the digital health ecosystem [90]. Importantly, in recognition of the ongoing digital divide globally, Minderoo Foundation worked collaboratively with in-country partners to adapt the program content for nondigital delivery, including through print resources, television and radio broadcasts, and group-based workshops. For those with access to mobile devices, the project would have been further enhanced by the provision of opportunities to build end user capability to use and derive benefit from the Thrive by Five app, as advised in the principles for digital development [91]. This approach appreciates that the digital divide extends beyond internet and device access but also relates to having the necessary digital skills to achieve the desired outcomes through engagement with the digital health program [92].

### Data Rather Than Assumptions: Clarifying the Target Audience

In-country partners and subject matter experts as well as parents and caregivers consistently questioned the applicability of the program for individuals from rural areas and with lower levels of education. While study findings are variable, there is not a consistent association between lower parental education and socioeconomic status and enrollment and participation in parenting programs [93,94]. Assumptions about interest in and relevance of parenting programs based on demographic factors risk excluding individuals who may benefit and, in turn, widening disparities in early child development. Indeed, learnings from the evaluation phase highlighted the significant potential for impact in rural communities where access to health services and culturally appropriate resources in local languages was often limited relative to urban settings [30]. Indeed, participants in rural areas frequently reported that they found the program made a meaningful difference in their day-to-day experience of child-rearing [30]. Importantly, these outcomes helped inform a shift in the identified target audience over time based on the greatest potential for impact, with a particular focus on individuals with lower levels of education living in rural areas.

### Prioritizing Sociocultural Context Through Language Translation Processes

Use of appropriate language and meticulous translation are fundamental to the success of international digital health initiatives. Literal word-for-word translations fail to convey the nuanced meaning of language, including tone, emotionality, word choice, and use of idioms [95]. As evidenced by our experience in Kyrgyzstan where a skilled translator was embedded as part of the in-country partner team, there are significant benefits to partnering with a translator, and most ideally a team of such individuals, throughout the R&D of the program. When research is conducted across boundaries of culture and context, it is critical that researchers do not assume knowledge or understanding. For example, our evaluation results indicated that participants found it challenging to comprehend some of the program content despite having translated the information into local languages and validated these translations with in-country partners [66]. Cocreating knowledge of language in collaboration with team-based translators is a critical way in which to embed context in R&D processes. Iteratively creating banks of words that could be misinterpreted or require clarification by individuals from differing cultures and contexts is one way in which to build knowledge among diverse teams and stakeholder groups and develop shared knowledge between translator teams [95]. In quantitative research, the Guidelines for Best Practice in Cross-Cultural Surveys recommends a team approach to translation using a TRAPD (translation, review, adjudication, pretesting, and documentation)-based approach [96]. It is recommended that the team include at least 2 independent translators and individuals with knowledge of the study design, field of research, and sociocultural context to ensure surveys convey the intended meaning, are easily understood by the target participants, and capture reliable data enabling meaningful comparisons between cultural groups.

Beyond the challenges of language translation, it is also important to recognize that surveys may not adequately capture the perspectives of individuals from diverse contexts, particularly in relation to potentially highly stigmatized topics such as mental health. Most mental health assessment surveys were originally developed in Western research contexts and rely on Western conceptualizations of mental illness that may not align with the experiences or beliefs of diverse populations. As evidenced by our research, this may lead to confusion, such as when required to respond on a 5-point Likert scale regarding changes in feelings of happiness (ie, 0=much less than I used to and 4=much more than I used to). The development of the Shona Symptom Questionnaire in Zimbabwe, the first Indigenous survey for the assessment of common mental health disorders, highlights the need to include local idioms of distress and concepts of illness relevant to local mental health practitioners when developing surveys for a specific audience [97]. Unfortunately, the availability of such meticulously developed tools for specific populations is rare. Rather than relying on Western-based survey instruments, even those that have been validated in diverse contexts, as we did in this study, flexible approaches may result in the collection of higher-quality data. Indeed, it has been shown that participants in LMICs often prefer to share their experiences and opinions verbally as opposed to having to record their thoughts using a Likert scale [98]. Considering participants’ preferred method of participation is critical.

Notably, it is our belief that a team approach is also well-suited to the interpretation of qualitative data. Indeed, our work underscores the importance of debrief sessions with translators to ongoingly engage in collaborative sense-making and develop knowledge of sociocultural context, particularly in relation to interpreting, reporting, and translating research outcomes. Furthermore, a team approach has the potential to minimize translator impacts on the research processes. As previously reported by Field et al [99], translators can shift power dynamics, omit details, mis- or underrepresent the perspectives of the participant, and deviate from established research protocols and methods. For example, in Uzbekistan, we found that having a second translator review interview and workshop recordings helped elucidate the impact of gender roles on translations, adding a new layer to the interpretation of the findings. While there is a lack of empirical evidence about best practice approaches to translation, available literature suggests that a team approach is more likely to identify errors and preserve intended meaning relative to single translator forwards-only translation, dual translator forward-back translation [100,101], or single translator.

### Limitations

This study has some limitations to note. The multicase study design was chosen retrospectively. Nevertheless, the mostly qualitative research methodologies used in the broader project were well-suited to this study design, particularly given the complex and deeply contextual nature of each individual case. The multicase approach enables a deep understanding of the program, including how this varies based on differing sociocultural and contextual conditions [47].

The research team was comprised of researchers from diverse cultural and ethnic backgrounds, including individuals originally from LMICs and those with Indigenous backgrounds; however, all research was conducted from outside the case countries, potentially emphasizing the perception of *distance* between researchers and participants. Importantly, the multicase study methodology by Stake [46] supports the practice of reflexivity as part of the research process, enabling reflection and shared learning about sociocultural and contextual factors interacting with and influencing R&D processes to inform adaptations as needed.

In addition, in-country fieldwork would have significantly enhanced this study; however, this was not feasible due to research funding as well as travel restrictions and safety concerns in some countries. Consequently, this research relied heavily on the use of technology for data collection, which likely contributed to the recruitment of participants from a higher socioeconomic group with greater access to digital technologies and from urban areas where digital infrastructure was more developed.

### Practical Implications and Conclusions

The learnings from this large-scale digital child-rearing program are extensive and multifaceted. The key lessons have been distilled in Textbox 3 with the aim of helping researchers consider potential strategies to facilitate successful R&D processes for their digital parenting projects.

**Ten key research lessons learned to inform future global digital parenting projects.**

- Meaningful and genuine collaboration is not built contractually. Dedicate time to understanding all parties’ goals and expectations for a project, consider the best ways of working together, explore and acknowledge power dynamics, and harness conflict to promote innovation.
- In-country partners’ established networks impacted recruitment of representative research samples and influenced program uptake and adoption. An expanded partnership base, including governmental and nongovernmental organizations, community leaders, and local change agents, is advised to broaden program reach and, in turn, health and social impacts.
- Recognize and acknowledge the power of governments to help or hinder digital parenting projects. Fostering relationships with governments as partners is often time-consuming and fraught with unstandardized processes and procedures, but failure to do so has the potential to undermine program success.
- A shared vision is critical for any digital parenting initiative. It creates a uniting sense of purpose, helps integrate diverse multidisciplinary and multiskilled teams, and motivates and inspires a drive for change and innovation.
- In digital health, it is imperative to identify your target audience before development begins to ensure the solution is needed and will be accessible and fit for purpose.
- Conducting a digital technology inventory to scope the information and communications technology infrastructure and policy and digital parenting marketplace before embarking on a project is paramount to ensure feasibility and desirability, enabling resources to be directed toward multichannel nondigital strategies when likely to be more impactful.
- Practicing cultural humility fosters a collective desire to learn about the experiences, beliefs, and needs of project stakeholders, both fostering collaboration and informing the ongoing iterative refinement of digital health interventions.
- Capacity building is bidirectional. It is important to recognize and value the experiences and expertise that both researchers and partner organizations bring to research projects to inform the feasibility and appropriateness of research methodology as well as data analysis and interpretation.
- Literal translations are insufficient. Embedding skilled translators in the team throughout the project will ensure they can balance the vision and objectives of the project with the language and terminology preferences (eg, formal vs informal language, use of local vernacular, and cultural understanding of key concepts) of the target audience.
- Complex digital parenting projects require time and flexibility to enable ongoing participation from critical stakeholders, adapt to dynamic sociocultural circumstances, and benefit from iterative feedback loops to promote scalability and sustainability.

Global parenting initiatives are inherently complex, and it is critical that sufficient time is allocated to build and foster collaborative partnerships based on respect, cultural understanding, and open communication. Furthermore, thoughtful and considered approaches are required to ensure the voices, experiences, values, and needs of the identified target audience are reflected in the program design, implementation strategies, and evaluation methods. It is only with these foundational elements in place that these types of projects can be successful. Importantly, the learnings identified in this research were derived specifically from an international child-rearing program; however, we have drawn from diverse research areas, including public health, global health, public policy and administration, change management, early childhood development, and digital health, to contextualize, interpret, and synthesize our findings. Therefore, while the learnings presented here are likely to be most informative for other research teams operating in the global digital parenting space, they are also likely to provide valuable insights for those conducting digital health research more generally.

## Appendix

Multimedia Appendix 1 Summary of research methodologies and data analyses.

Multimedia Appendix 2 Co-design workshop agenda.

Multimedia Appendix 3 Impact evaluation interview schedule.

Multimedia Appendix 4 Impact evaluation workshop agenda.

Multimedia Appendix 5 Process evaluation interview schedule.

## Abbreviations

DRC: Democratic Republic of the Congo
HIC: high-income country
HREC: Human Research Ethics Committee
ICT: information and communications technology
LMICs: low- and middle-income countries
MAPECE: Malaysian Association of Professional Early Childhood Educators
MRC: Medical Research Council
PNG: Papua New Guinea
R&D: research and development
REDCap: Research Electronic Data Capture
TRAPD: translation, review, adjudication, pretesting, and documentation
WEIRD: Western, educated, industrialized, rich, and democratic

## References

[1] Transforming our world: the 2030 agenda for sustainable development. United Nations.

[2] Black MM, Walker SP, Fernald LC, Andersen CT, DiGirolamo AM, Lu C, McCoy DC, Fink G, Shawar YR, Shiffman J, Devercelli AE, Wodon QT, Vargas-Barón Emily, Grantham-McGregor S, Lancet Early Childhood Development Series Steering Committee (2017). Early childhood development coming of age: science through the life course. Lancet.

[3] Engle PL, Black MM, Behrman JR, Cabral de Mello M, Gertler PJ, Kapiriri L, Martorell R, Young ME (2007). Strategies to avoid the loss of developmental potential in more than 200 million children in the developing world. Lancet.

[4] Engle PL, Fernald LC, Alderman H, Behrman J, O'Gara C, Yousafzai A, de Mello MC, Hidrobo M, Ulkuer N, Ertem I, Iltus S, Global Child Development Steering Group (2011). Strategies for reducing inequalities and improving developmental outcomes for young children in low-income and middle-income countries. Lancet.

[5] Barlow J, Coren E, Stewart-Brown S (2002). Meta-analysis of the effectiveness of parenting programmes in improving maternal psychosocial health. Br J Gen Pract.

[6] Barlow J, Parsons J, Stewart-Brown S (2005). Preventing emotional and behavioural problems: the effectiveness of parenting programmes with children less than 3 years of age. Child Care Health Dev.

[7] Barlow J, Smailagic N, Huband N, Roloff V, Bennett C (2014). Group-based parent training programmes for improving parental psychosocial health. Cochrane Database Syst Rev.

[8] Jeong J, Franchett EE, Ramos de Oliveira CV, Rehmani K, Yousafzai AK (2021). Parenting interventions to promote early child development in the first three years of life: a global systematic review and meta-analysis. PLoS Med.

[9] Mingebach T, Kamp-Becker I, Christiansen H, Weber L (2018). Meta-meta-analysis on the effectiveness of parent-based interventions for the treatment of child externalizing behavior problems. PLoS One.

[10] Rayce SB, Rasmussen IS, Klest SK, Patras J, Pontoppidan M (2017). Effects of parenting interventions for at-risk parents with infants: a systematic review and meta-analyses. BMJ Open.

[11] World Health Organization, United Nations Children's Fund, World Bank Group (2018). Nurturing care for early childhood development. World Health Organization.

[12] Britto PR, Lye SJ, Proulx K, Yousafzai AK, Matthews SG, Vaivada T, Perez-Escamilla R, Rao N, Ip P, Fernald LC, MacMillan H, Hanson M, Wachs TD, Yao H, Yoshikawa H, Cerezo A, Leckman JF, Bhutta ZA, Early Childhood Development Interventions Review Group‚ for the Lancet Early Childhood Development Series Steering Committee (2017). Nurturing care: promoting early childhood development. Lancet.

[13] International Telecommunication Union, World Health Organization (2020). Digital health platform handbook: building a digital information infrastructure (‎infostructure)‎ for health. World Health Organization.

[14] The Inclusive Internet Index Five-year lookback report. Economist Impact.

[15] Featherstone D, Ormond-Parker L, Ganley L, Thomas J, Parkinson S, Hegarty K, Kennedy J, Holcombe-James I, Valenta L, Hawkins L (2023). Mapping the digital gap: 2023 outcomes report. Analysis & Policy Observatory.

[16] (2023). Measuring digital development: facts and figures 2023. International Telecommunication Union.

[17] Canário AC, Byrne S, Creasey N, Kodyšová E, Kömürcü Akik B, Lewandowska-Walter A, Modić Stanke K, Pećnik N, Leijten P (2022). The use of information and communication technologies in family support across Europe: a narrative review. Int J Environ Res Public Health.

[18] Thongseiratch T, Leijten P, Melendez-Torres GJ (2020). Online parent programs for children's behavioral problems: a meta-analytic review. Eur Child Adolesc Psychiatry.

[19] Leijten P, Rienks K, Groenman AP, Anand M, Kömürcü Akik B, David O, Kızıltepe R, Thongseiratch T, Catarina Canário A (2024). Online parenting support: meta-analyses of non-inferiority and additional value to in-person support. Child Youth Serv Rev.

[20] Cai Q, Buchanan G, Simenec T, Lee SK, Basha SA, Gewirtz AH (2024). Enhancing engagement in parenting programs: a comparative study of in-person, online, and telehealth formats. Child Youth Serv Rev.

[21] Metzler CW, Sanders MR, Rusby JC, Crowley RN (2012). Using consumer preference information to increase the reach and impact of media-based parenting interventions in a public health approach to parenting support. Behav Ther.

[22] Weisenmuller C, Hilton D (2021). Barriers to access, implementation, and utilization of parenting interventions: considerations for research and clinical applications. Am Psychol.

[23] Craig P, Dieppe P, Macintyre S, Michie S, Nazareth I, Petticrew M (2008). Developing and evaluating complex interventions: the new Medical Research Council guidance. BMJ.

[24] Skivington K, Matthews L, Simpson SA, Craig P, Baird J, Blazeby JM, Boyd KA, Craig N, French DP, McIntosh E, Petticrew M, Rycroft-Malone J, White M, Moore L (2021). A new framework for developing and evaluating complex interventions: update of Medical Research Council guidance. BMJ.

[25] Skivington K, Craig N, Craig P, Rycroft-Malone J, Matthews L, Simpson SA, Moore L (2024). Introducing the revised framework for developing and evaluating complex interventions: a challenge and a resource for nursing research. Int J Nurs Stud.

[26] Scheidecker G, Chaudhary N, Keller H, Mezzenzana F, Lancy DF (2023). “Poor brain development” in the global South? Challenging the science of early childhood interventions. Ethos.

[27] Bornstein MH, Cheah CS, Rubin KH, Chung OB (2006). The place of "culture and parenting" in the ecological contextual perspective on developmental science. Parenting Beliefs, Behaviors, and Parent-Child Relations: A Cross-cultural Perspective.

[28] LaMonica HM, Crouse JJ, Song YJ, Alam M, Wilson CE, Hindmarsh G, Yoon A, Boulton KA, Ekambareshwar M, Loblay V, Troy J, Torwali M, Guastella AJ, Banati RB, Hickie IB (2023). Developing culturally appropriate content for a child-rearing app to support young children's socioemotional and cognitive development in Afghanistan: co-design study. JMIR Form Res.

[29] Bornstein MH (2012). Cultural approaches to parenting. Parent Sci Pract.

[30] Loblay V, Ekambareshwar M, Naderbagi A, Song YJ, Ford M, Zahed I, Yoon A, Hickie IB, LaMonica HM (2023). Enhancing equitable engagement for digital health promotion: lessons from evaluating a childrearing app in Indonesia. Digit Health.

[31] Lupton D (2017). Digital Health: Critical and Cross-Disciplinary Perspectives.

[32] Craig P, Di Ruggiero E, Frohlich KL, Mykhalovskiy E, White M (2018). Taking Account of Context in Population Health Intervention Research: Guidance for Producers, Users and Funders of Research.

[33] Naderbagi A, Loblay V, Zahed IU, Ekambareshwar M, Poulsen A, Song YJ, Ospina-Pinillos L, Krausz M, Mamdouh Kamel M, Hickie IB, LaMonica HM (2024). Cultural and contextual adaptation of digital health interventions: narrative review. J Med Internet Res.

[34] Poulsen A, Hickie IB, Alam M, Crouse JJ, Ekambareshwar M, Loblay V, Song YJ, LaMonica HM (2024). Overcoming barriers to mHealth co-design in low- and middle-income countries: a research toolkit. Inf Technol Dev.

[35] Resnicow K, Soler R, Braithwaite RL, Ahluwalia JS, Butler J (2000). Cultural sensitivity in substance use prevention. J Community Psychol.

[36] Hawe P, Shiell A, Riley T (2004). Complex interventions: how "out of control" can a randomised controlled trial be?. BMJ.

[37] Crowe S, Cresswell K, Robertson A, Huby G, Avery A, Sheikh A (2011). The case study approach. BMC Med Res Methodol.

[38] Paparini S, Papoutsi C, Murdoch J, Green J, Petticrew M, Greenhalgh T, Shaw SE (2021). Evaluating complex interventions in context: systematic, meta-narrative review of case study approaches. BMC Med Res Methodol.

[39] Murdoch J, Paparini S, Papoutsi C, James H, Greenhalgh T, Shaw SE (2023). Mobilising context as complex and dynamic in evaluations of complex health interventions. BMC Health Serv Res.

[40] Star SL, Griesemer JR (1989). Institutional ecology, `translations' and boundary objects: amateurs and professionals in Berkeley's museum of vertebrate zoology, 1907-39. Soc Stud Sci.

[41] (2023). Classification of digital interventions, services and applications in health: a shared language to describe the uses of digital technology for health. World Health Organization.

[42] Bornstein MH, Cluver L, Deater-Deckard K, Hill NE, Jager J, Krutikova S, Lerner RM, Yoshikawa H (2022). The future of parenting programs: I design. Parenting.

[43] Crouse JJ, LaMonica HM, Song YJ, Boulton KA, Rohleder C, DeMayo MM, Wilson CE, Loblay V, Hindmarsh G, Stratigos T, Krausz M, Foo N, Teo M, Hunter A, Guastella AJ, Banati RB, Troy J, Hickie IB (2023). Designing an app for parents and caregivers to promote cognitive and socioemotional development and well-being among children aged 0 to 5 years in diverse cultural settings: scientific framework. JMIR Pediatr Parent.

[44] LaMonica HM, Crouse JJ, Song YJ, Alam M, Ekambareshwar M, Loblay V, Yoon A, Cha G, Wilson C, Sweeney-Nash M, Foo N, Teo M, Perhirin M, Troy J, Hickie IB (2022). Developing a parenting app to support young children's socioemotional and cognitive development in culturally diverse low- and middle-income countries: protocol for a co-design study. JMIR Res Protoc.

[45] LaMonica HM, Song YJ, Loblay V, Ekambareshwar M, Naderbagi A, Zahed IU, Troy J, Hickie IB (2024). Promoting social, emotional, and cognitive development in early childhood: a protocol for early valuation of a culturally adapted digital tool for supporting optimal childrearing practices. Digit Health.

[46] Stake RE (1995). The Art of Case Study Research.

[47] Stake R (2006). Multiple Case Study Analysis.

[48] Harris PA, Taylor R, Minor BL, Elliott V, Fernandez M, O'Neal L, McLeod L, Delacqua G, Delacqua F, Kirby J, Duda SN (2019). The REDCap consortium: building an international community of software platform partners. J Biomed Inform.

[49] Braun V, Clarke V (2006). Using thematic analysis in psychology. Qual Res Psychol.

[50] Guo M, Morawska A, Filus A (2017). Validation of the parenting and family adjustment scales to measure parenting skills and family adjustment in Chinese parents. Meas Eval Counsel Dev.

[51] Sanders MR, Morawska A, Haslam DM, Filus A, Fletcher R (2014). Parenting and Family Adjustment Scales (PAFAS): validation of a brief parent-report measure for use in assessment of parenting skills and family relationships. Child Psychiatry Hum Dev.

[52] Sumargi A, Filus A, Morawska A, Sofronoff K (2017). The Parenting and Family Adjustment Scales (PAFAS): an Indonesian validation study. J Child Fam Stud.

[53] Črnčec R, Barnett B, Matthey S (2019). Karitane Parenting Confidence Scale (KPCS) manual v.1. Karitane.

[54] Usui Y, Haruna M, Shimpuku Y (2020). Validity and reliability of the Karitane Parenting Confidence Scale among Japanese mothers. Nurs Health Sci.

[55] Brooke J (1996). SUS: a 'quick and dirty' usability scale. Usability Evaluation In Industry.

[56] Sauro J, Lewis JR (2012). Quantifying the User Experience: Practical Statistics for User Research.

[57] Lewis JR (2018). The system usability scale: past, present, and future. Int J Hum Comput Interact.

[58] Alam M, Hickie IB, Poulsen A, Ekambareshwar M, Loblay V, Crouse J, Hindmarsh G, Song YJ, Yoon A, Cha G, Wilson C, Sweeney-Nash M, Troy J, LaMonica HM (2023). Parenting app to support socio-emotional and cognitive development in early childhood: iterative codesign learnings from nine low-income and middle-income countries. BMJ Open.

[59] LaMonica HM, Loblay V, Khan Q, Hindmarsh G, Song YJ, Ekambareshwar M, Ospina-Pinillos L, Hickie IB (2025). Parental emotional adjustment as a primary target for parenting programs: a cross-sectional study. BMC Psychol.

[60] Australia's national standards and certifying authority for translators and interpreters. National Accreditation Authority for Translators and Interpreters.

[61] Harris PA, Taylor R, Thielke R, Payne J, Gonzalez N, Conde JG (2009). Research electronic data capture (REDCap)--a metadata-driven methodology and workflow process for providing translational research informatics support. J Biomed Inform.

[62] (2023). Nvivo (Version 14). Lumivero.

[63] West MA, Johnson DA, Beyerlein ST (2000). Reflexivity, revolution and innovation in work teams. Advances in Interdisciplinary Study of Work Teams: Product Development Teams.

[64] Widmer PS, Schippers M, West MA (2009). Recent developments in reflexivity research: a review. Psychol Everyday Activity.

[65] Cayir E, Felder TM, Nkwonta CA, Jackson JR, Dawson R (2022). Discovering new connections: insights from individual and collective reflexivity in a mixed methods study. Int J Qual Methods.

[66] Khan Q, Hickie IB, Loblay V, Ekambareshwar M, Zahed IU, Naderbagi A, Song YJ, LaMonica HM (2025). Psychometric evaluation of the System Usability Scale in the context of a childrearing app co-designed for low- and middle-income countries. Digit Health.

[67] Garner SL, Koch H, George CE, Hitchcock J, Norman G, Green G, Young P, Mahid Z (2021). Cross cultural team collaboration: integrating cultural humility in mHealth development and research. Inform Health Soc Care.

[68] Google Play Asianparent: Pregnancy and baby. Google Play.

[69] Brinkerhoff JM (2002). Government–nonprofit partnership: a defining framework. Public Admin Dev.

[70] Gazley B, Brudney JL (2007). The purpose (and perils) of government-nonprofit partnership. Nonprofit Volunt Sect Q.

[71] Iyamu I, Gómez-Ramírez O, Xu AX, Chang HJ, Watt S, Mckee G, Gilbert M (2022). Challenges in the development of digital public health interventions and mapped solutions: findings from a scoping review. Digit Health.

[72] Klijn EH, Hodge G, Greve C (2010). Public private partnerships: deciphering meaning, message and phenomenon. International Handbook of PPP.

[73] Bryson JM, Crosby BC, Stone MM (2006). The design and implementation of cross‐sector collaborations: propositions from the literature. Public Adm Rev.

[74] Person AE, Strong DA, Furgeson J, Berk JA (2009). Maximizing the value of philanthropic efforts through planned partnerships between the U.S. government and private foundations. U.S. Department of Health and Human Services.

[75] Fransman J, Hall B, Hayman R, Narayanan P, Newman K, Tandon R (2021). Beyond partnerships: embracing complexity to understand and improve research collaboration for global development. Can J Dev Stud.

[76] Coleman PT, Vallacher RR, Bartoli A, Nowak A, Bui-Wrzosinska L, Körppen D, Ropers N, Giessmann HJ (2011). Navigating the landscape of conflict: applications of dynamical systems theory to protracted social conflict. The Non-Linearity of Peace Processes. Theory and Practice of Systemic Conflict Transformation.

[77] Fransman J, Newman K (2019). Rethinking research partnerships: evidence and the politics of participation in research partnerships for international development. J Int Dev.

[78] Loblay V, Conte KP, Grøn S, Green A, Innes-Hughes C, Milat A, Persson L, Williams M, Mitchell J, Hawe P (2021). The weight of words: co-analysis of thick ethnographic description and "friction" as methodological strategies in a health policy research partnership. Qual Health Res.

[79] ten Have S, ten Have W, Huijsmans AB, Otto M (2016). Reconsidering Change Management: Applying Evidence-Based Insights in Change Management Practice.

[80] Stouten J, Rousseau DM, De Cremer D (2018). Successful organizational change: integrating the management practice and scholarly literatures. Acad Manag Ann.

[81] Doten-Snitker K, Margherio C, Litzler E, Ingram E, Williams J (2020). Developing a shared vision for change: moving toward inclusive empowerment. Res High Educ.

[82] Freeth R, Caniglia G (2019). Learning to collaborate while collaborating: advancing interdisciplinary sustainability research. Sustain Sci.

[83] Hargrave TJ, Van De Ven AH (2006). A collective action model of institutional innovation. Acad Manag Rev.

[84] Batley R, Rose P (2011). Analysing collaboration between non‐governmental service providers and governments. Public Admin Dev.

[85] Almog-Bar M, Zychlinski E (2012). A façade of collaboration. Public Manag Rev.

[86] Mundy K, Haggerty M, Sivasubramaniam M, Cherry S, Maclure R (2011). Civil society, basic education, and sector-wide aid: insights from sub-Saharan Africa. Dev Pract.

[87] International Telecommunication Union, United Nations Educational, Scientific and Cultural Organization (2022). The state of broadband 2022: accelerating broadband for new realities. Broadband Commission.

[88] (2020). Digital transformation policy 2020. Papua New Guinea Department of Information and Communications Technology.

[89] National right to information policy 2020-2030. Papua New Guinea Department of Information and Communications Technology.

[90] (2019). Recommendations on digital interventions for health system strengthening: WHO guideline. World Health Organization.

[91] Principles for digital development homepage. Principles for Digital Development.

[92] Livingstone S, Mascheroni G, Stoilova M (2021). The outcomes of gaining digital skills for young people’s lives and wellbeing: a systematic evidence review. New Media Soc.

[93] Dumas JE, Nissley-Tsiopinis J, Moreland AD (2006). From intent to enrollment, attendance, and participation in preventive parenting groups. J Child Fam Stud.

[94] Hackworth NJ, Matthews J, Westrupp EM, Nguyen C, Phan T, Scicluna A, Cann W, Bethelsen D, Bennetts SK, Nicholson JM (2018). What influences parental engagement in early intervention? Parent, program and community predictors of enrolment, retention and involvement. Prev Sci.

[95] Haldane V, Li BP, Ge S, Huang JZ, Huang H, Sadutshang L, Zhang Z, Pasang P, Hu J, Wei X (2022). Exploring the translation process for multilingual implementation research studies: a collaborative autoethnography. BMJ Glob Health.

[96] Mohler P, Dorer B, Jong J, Hu M (2016). Translation: overview. Guidelines for best practice in cross-cultural surveys. Survey Research Center, Institute for Social Research, University of Michigan.

[97] Patel V, Simunyu E, Gwanzura F, Lewis G, Mann A (1997). The Shona Symptom Questionnaire: the development of an indigenous measure of common mental disorders in Harare. Acta Psychiatr Scand.

[98] Tennyson RL, Kemp CG, Rao D (2016). Challenges and strategies for implementing mental health measurement for research in low-resource settings. Int Health.

[99] Field RS, Barns A, Chung D, Fleay C (2021). Messiness in international qualitative interviewing: what I did, what I didn’t do, and a little bit about why. Qual Soc Work.

[100] Forsyth BH, Kudela MS, Levin K, Lawrence D, Willis GB (2007). Methods for translating an English-language survey questionnaire on tobacco use into Mandarin, Cantonese, Korean, and Vietnamese. Field Methods.

[101] Vujcich D, Roberts M, Gu Z, Kao SC, Lobo R, Mao L, Oudih E, Phoo NN, Wong H, Reid A (2021). Translating best practice into real practice: methods, results and lessons from a project to translate an English sexual health survey into four Asian languages. PLoS One.

