# Supplementary material for: Top 10 Research Lessons Learned From a Digital Child-Rearing Program in Low- and Middle-Income Countries: Multicase Study
Source: J Med Internet Res. 2025 Jul 29;27:e65705. doi: 10.2196/65705 (PMC12344384; doi:10.2196/65705)
Supplement: Multimedia Appendix 1 [file jmir_v27i1e65705_app1.docx]

# Multimedia Appendix 1. Summary of research methodologies and data analyses

## Scientific framework

As explained in detail in Crouse et al. [45], the content for the Thrive by Five International Program is underpinned by a scientific framework that highlights key neurobiological systems that support a child’s socioemotional and cognitive development. The Thrive by Five content is designed to target these key systems behaviourally to support healthy early childhood development. The five systems and their main functions include (1) the stress response system, which creates a hormonal response to stress (prolonged activation of the stress response system is associated with negative emotional, behavioural, and physical health outcomes); (2) the oxytocin system, which regulates social, behavioural, and emotional processes (eg, smiling, attention to eye gaze, and breastfeeding), of which many are fundamentally important for early child-caregiver bonds and other social bonds; (3) the learning system, which assigns value to objects and behaviours (in childhood, this is fundamental for motivation creation, social behaviours, and associative learning); (4) the fear-arousal-memory system, which encodes and maintains memories of fearful stimuli and the contexts in which they are experienced; and (5) the circadian system, which orchestrates the daily rhythmic timing of almost all physiological processes and behaviours (eg, sleep and wakefulness, appetite, mood, and cognitive function).

## Literature review

For each participating country, the research team began by conducting a comprehensive literature review of each country’s historical and sociocultural context in collaboration with academics with expert knowledge relevant to the R&D context [28]. The literature review summarised information from a variety of published literature, including government reports, journal articles, and textbooks. While a dedicated pro forma was used to guide the search, there was considerable variability in the quality and overall availability of relevant literature across the different countries. That said, we focused on reviewing information about: 1) the cultural practices impacting childrearing (i.e., roles of parents, siblings, extended family, community, and other caregivers; position of the child in the family, extended family and community; maternal health, childbirth, and early childhood health); 2) recognised collectivist and individualist approaches to childrearing (i.e., at the country level; at the subgroup level); 3) gender roles of parents, grandparents, caregivers, and children (i.e., social status of women; childrearing and household responsibilities; maternal and early childhood education; workforce participation; child and consanguineous marriage as relevant); 4) historic, economic, social, religious, ecological, or other transgenerational factors (i.e., impacts of colonisation; changing status of women; industrialisation and/or migrant work; urbanisation; civil disruptions or conflict; impacts of religion and religious practices; climate change and natural disasters; recognition of disadvantaged or excluded subgroups; 5) Any other cultural factors that may be expected to influence parenting practices (i.e., traditional practices, rituals, and ceremonies that support an enriched social environment for early childhood development; approaches to preschool education; languages spoken; cultural approaches to behaviour management; cultural meaning of play. Importantly, the accuracy and completeness of the literature review was validated by the in-country partner and subject matter experts prior to embarking on any further research. The learnings from the literature review informed preliminary content development for the Program.

## Co-design methods and data analysis

In each country, the content was iteratively developed, tested, and refined in collaboration with parents, child caregivers (e.g., grandparents, aunts and uncles), and subject matter experts in accordance with a standardised co-design protocol [46]. Specifically, in each country, on average, a series of four workshops were conducted with parents and caregivers and two workshops were conducted with subject matter experts. The workshops were co-facilitated by members of the research team (HL, AP, and MA) in collaboration with Minderoo Foundation and a member of the research team from the in-country partner. Workshops ranged in length from 2-4 hours. Using prompted discussion questions, participants actively contributed to the development and refinement of the content, informing its cultural appropriateness, contextual relevance, and alignment with user needs. The overarching agenda for these workshops has been published elsewhere [46]; however, example questions are included in Textbox 1.

Textbox 1. Example questions used to prompt discussion in co-design workshops.

- What information would parents want to help care for children aged 0-5 years?
- When you think back to being a first-time parent (depending on the number of children), what did you want to know about raising a child?
- What values or morals do you want to instil in children?
- What skills do you want your child to learn by the age of 5?
- How do young children socialise with other children in the community?
- How do you manage tantrums and big emotions of young children?
- What is the role of television and mobile phones in children’s day-to-day activities?

Interpretation of the qualitative data from the workshops followed established thematic techniques (i.e., inductive reasoning) [53]. All raw data was reviewed and checked across all participants by senior researchers (AP, MA, HL) to develop a coding framework specific to a country’s sociocultural context, outlining all key concepts. Subsequently, data was coded in NVivo software [54] using this framework by two researchers (AP, MA). The coding adhered to an established iterative process of reading, coding, and exploring the pattern and content of coded data, followed by reflection and discussion to reach consensus. The key lessons learned from the co-design research for each case were synthesised in an outcomes report to build understanding of the sociocultural context, document feedback on the preliminary content, and examine potential issues related to user experience. Each report included specific recommendations as to how to improve existing Collective Actions, and what, if any, new Collective Actions should be developed to fill gaps identified by participants, both of which were actioned by the research team. Particular attention was paid to ensure the localisation of the content was culturally appropriate, including the use of language, terminology, and examples of local content (e.g., songs, dances, games, children’s stories). The finalisation of a fully revised library of content, including validation by the in-country partner and subject matter experts, concluded this phase of the research.

## Outcomes and process evaluation methods and data analysis

Following implementation of the Program in most countries, the research team conducted a mixed-methods study with the primary aim of evaluating the impact of the co-designed Thrive by Five app and its content on parent and child caregiver knowledge, behaviours, attitudes, and confidence, as well as the connection between the child and their parents, family, community and culture. In addition, the study aimed to explore the cultural and contextual factors influencing Program engagement; how engagement with the content influenced family (e.g., between a child and a parent, a wife and a husband, a mother and mother-in-law) and community relationships (e.g., community leaders as models of new behaviours); the cultural appropriateness and relevance of the content; the quality, usability, and acceptability of the app and content; and the processes that occurred prior to and during implementation of the Thrive by Five International Program that influenced awareness, uptake, adoption and engagement.

The protocol for this mixed-methods evaluation study has been published elsewhere [47]; therefore, a summary of the methodology is provided here. Parents and child caregivers were able to provide their feedback on the Program via a quantitative survey or through qualitative semi-structured interviews and workshops. The survey was comprised of three standardised questionnaires, all of which have been used in cross-cultural contexts, including: the Parenting and Family Adjustment Scales, a 30-item validated tool assessing changes in childrearing practices and behaviours following participation in a parenting program [97-99]; the Parenting Confidence Scale, a 15-item tool to measure how confident a parent is in their parenting skills [100-101]; and the System Usability Scale, a 10-item measure of app usability and acceptability [102-104]. Additional survey questions were designed specifically for this study and measured self-reported app usage and satisfaction, changes in a child’s connection with their parent(s), family, community, and culture, and knowledge of early childhood development [47].

To explore the effects of Thrive by Five in more depth, parents and child caregivers also had the opportunity to participate in one-on-one semi-structured interviews or group-based workshops conducted by a member(s) of the research team (VL and ME). Interview schedules and workshop agendas (refer to [47] for details) were designed to be flexible to facilitate exploration of the variability in uptake and impact based on cultural and contextual factors.

To examine the processes that supported the co-design, implementation, promotion and dissemination, and evaluation activities, the researchers (VL and ME) also conducted opportunistic semi-structured and conversational interviews with participants who had been actively involved in country-specific R&D activities. This included members of the Minderoo Foundation team, in-country partners from the research site, subject matter experts, and other key stakeholders (e.g., translators). These interviews were designed to explore: the system change processes supporting and shaped by the Program, how partnerships were influenced by sociocultural and contextual factors, and challenges experienced by collaborators when establishing partnerships and implementing the Program.

As described in detail in LaMonica et al. [47], quantitative data was analysed using a mix of descriptive and interferential statistics, tests for variability, and correlations and multivariate regression analyses. Qualitative data was analysed and interpreted using established thematic techniques similar to those described previously. In this instance, senior researchers (VL and ME) coded all data in NVivo developed a coding framework to explore key concepts relevant to the evaluation objectives, including examining outcomes at the level of the individual, family, community, and broader social and health systems. An evaluation analysis report was produced to summarise the quantitative and qualitative outcomes in relation to changes in parental knowledge, behaviours, and attitudes towards parenting practices to support early childhood development; parental confidence in parenting skills; the usability and acceptability of the Thrive by Five app; and other unique findings relative to sociocultural context.

## References

1. Guo M, Morawska A and Filus A. (2017). Validation of the parenting and family adjustment scales to measure parenting skills and family adjustment in Chinese parents. Measurement and Evaluation in Counselling and Development, 50(3): 139–154. [https://doi.org/10.1080/07481756.2017.1327290](https://psycnet.apa.org/doi/10.1080/07481756.2017.1327290)
2. Sanders MR, Morawska A, Haslam DM, Filus A, Fletcher R. (2014). Parenting and family adjustment scales (PAFAS): validation of a brief parent-report measure for use in assessment of parenting skills and family relationships. Child Psychiatry Hum Dev 2014; 45: 255–272. <https://doi.org/10.1007/s10578-013-0397-3>.
3. Sumargi A, Filus A, Morawska A, Sofronoff K. (2018). The parenting and family adjustment scales (PAFAS): an Indonesian validation study. J Child Fam Stud, 27: 756–770. <https://doi.org/10.1007/s10826-017-0926-y>
4. Črnčec R, Barnett B and Matthey S. Karitane parenting confidence scale: manual. Sydney South West Area Health Service. Sydney: Australia, 2008. URL: <https://plct.org.uk/wp-content/uploads/2019/01/karitane-parenting-confidence-scale-manual-copy.pdf>
5. Usui Y, Haruna M and Shimpuku Y. (2020). Validity and reliability of the Karitane parenting confidence scale among Japanese mothers. Nurs Health Sci, 22: 205–211. <https://doi.org/10.1111/nhs.12633>
6. Brooke J. (1996). SUS-A quick and dirty usability scale. Usability Evaluat Industry, 189: 4–7. ISBN: 9780429157011.
7. Sauro J and Lewis JR. Quantifying the user experience: practical statistics for user research. Burlington, MA, USA: Morgan Kaufman, 2012.
8. Lewis JR. (2018). The system usability scale: past, present, and future. Int J Hum-Comput Int, 7: 577–590. [https://doi.org/10.1080/10447318.2018.1455307](https://psycnet.apa.org/doi/10.1080/10447318.2018.1455307)
