# Supplementary material for: Top 10 Research Lessons Learned From a Digital Child-Rearing Program in Low- and Middle-Income Countries: Multicase Study
Source: J Med Internet Res. 2025 Jul 29;27:e65705. doi: 10.2196/65705 (PMC12344384; doi:10.2196/65705)
Supplement: Multimedia Appendix 3 [file jmir_v27i1e65705_app3.docx]

**Multimedia Appendix 3: Impact Evaluation Semi-Structured Interview**

| 1 | Family Story | - Can you tell me a bit about your family and why you decided to use Thrive by Five? - *How many children under the age of 5?* - *What is your relationship to these children?* - *Where would you usually go for parenting guidance or information? Have you tried other parenting apps?* |
| --- | --- | --- |
| 2 | Use of Thrive by Five | - How did you learn about Thrive by Five? - *Roughly how long have you been using the app?* - Can you tell me about when you use the app? - *How often do you use it?* - Do you talk about it with others in the family or do they use it with you? What do they think of Thrive by Five? - Were there any competing issues or influencers that enabled you to actively use the Thrive by Five app or prevented you from actively using the app? |
| 3 | Thrive by Five Content | - Which activities from Thrive by Five have worked well in your family? Were there activities that didn’t work well? - How did you find the content in Thrive by Five? Was it relevant for your day-to-day interactions with young children? |
| 4 | Confidence and Connection | - Has Thrive by Five changed the way you interact with your child? How so? - Has using Thrive by Five improved your sense of confidence as a caregiver? In what ways? - How did the scientific content make you feel about trying new activities and making decisions? - Do you feel better prepared to make decisions about what is best for the children in your family since using the app? - Can you give an example of how a time when you used something you learned in the app to make a decision? - Did the activities in Thrive by Five affect your relationships with the children you care for? In what ways? Were any activities particularly helpful for connecting with the children? - Did anyone else in the family or broader community improve their connection with the child through the Thrive by Five activities? How so? |
| 5 | Knowledge gain | - What did you learn from Thrive by Five? Can you provide some specific examples? - Was the scientific content in Thrive by Five easy to understand? How did it affect your understanding of childhood development? - Was the content consistent with what you have learned about parenting and child development from other caregivers, doctors or health professionals, or the media? - How has the new knowledge impacted on your parenting? Can you provide an example? - How has the new knowledge impacted on your child? Can you give an example (eg. behaviour emotion regulation, mood, etc)? - Do you think you have a better understanding of how you can influence your child’s future? How has this impacted your attitude towards parenting? - What new knowledge has had the biggest impact on your relationship with the child? - Have you changed any of the practices / behaviours as a result of using the Thrive by Five app? If you have changed practices / behaviours, which practices have you changed? And what made you adapt the new behaviour / practice? - Is there additional information you wish had been in the app? Are there gaps in the content or information that you think would help you be the best parent you can be to your child? |
| 6 | Cultural Impact | - Thrive by Five is an app that is being used in many countries across the world. Did you find the app was supportive for raising children in your culture? - Did any activities challenge values that are important in your culture? If so, which ones? - How do you feel Thrive by Five fits with traditional knowledge about raising children? - Has anything changed in terms of how people in your community think about raising children as a result of Thrive by Five? - Do you feel that Thrive by Five has been good for your family and/or the broader community? - Do you feel that the knowledge from Thrive by Five is compatible with sharing cultural knowledge and reinforcing connections with language, land and country? How so? |
| 7 | Demographics | Before we finish up, I just need a few details from you:   - Can you tell me how old you are? - Can you tell me what was your highest level of education? Prompts – primary school, high school, diploma, degree etc. - How would you describe your current employment status (e.g., full-time employment, home duties, etc.)? - Note, sex of the participant will be observed by the interviewer. |
