# Supplementary material for: Top 10 Research Lessons Learned From a Digital Child-Rearing Program in Low- and Middle-Income Countries: Multicase Study
Source: J Med Internet Res. 2025 Jul 29;27:e65705. doi: 10.2196/65705 (PMC12344384; doi:10.2196/65705)
Supplement: Multimedia Appendix 5 [file jmir_v27i1e65705_app5.docx]

**Multimedia Appendix 5: Collaborators’ (site-specific research team, local subject matter experts, and other key stakeholders) perceptions - semi-structured interview**

| 1 | Role and responsibilities | - Can you tell us a bit about your organisation and   your usual role?   - How long have you been working in the organisation? - What experience do you have in working with parents and early childhood development? |
| --- | --- | --- |
| 2 | Partnership story | - In what ways have you been involved with the Thrive by Five app? - How did your organisation become involved with the Thrive by Five app? - Can you tell us about your experiences with the implementation of the app within your country?   *Prompt to describe planning process if it doesn’t come up*   - What role has your organisation played in the implementation of the app? - Were there other people or organisations that assisted with the implementation of the Thrive by Five app? Can you describe their role/involvement? - Has your involvement with Thrive by Five led to new partnerships or relationships with other organisations or colleagues in your country?   *Prompt to describe* |
| 3 | Value add and fit with existing activity | - How does Thrive by Five add to what you are already doing in this area? Why is that important? - Did you learn anything new from your engagements with the Thrive by Five app? Has this changed how you approach childhood development concepts? - What other resources do you provide parents to support early childhood development? - What resources are available to people with low literacy, or those who don’t have access to the internet or mobile phones? How does Thrive by Five compare to these? |
| 4 | Dissemination experiences | - Do you use Thrive by Five as part of your professional practice? How so? - Do you suggest parents/caregivers try the app?   *If yes:* Can you describe how parents respond to the Thrive by Five app?   - Were some parents/caregivers hesitant to sign up to the app? Why? - Has anyone told you about their experience of using the app? *Prompt to describe* |
| 5 | Implementation context | - Based on your experience with implementation of the Thrive by Five app, what were some of the key factors that influenced the implementation of the app within your country? - Were there training processes that supported the implementation of the app? If so, can you describe your experience of training? - Were there any other projects/activities that took place at the same time as the implementation of the Thrive by Five app? Did they facilitate and/or inhibit implementation of the app? |
| 4 | Evaluation | Please take a few minutes to think about any and all changes that may have happened since the Thrive by Five App was launched.   - For users and community members: Changes related to using the Thrive by Five App - For providers: Changes related to including the Thrive by Five App in your services   From your point of view, describe a story that best illustrates the most significant change that you have experienced as a result of the Thrive by Five app being offered in your community or being used in your professional practice.   - Why is this story significant for you?   Were you involved in the testing and design phases of the app? Describe your experience of this process and what was your contribution?  Did your organisation set goals in relation to implementation of the Thrive by Five app?  *Prompt to describe*   - To what extent have these goals been achieved, or are expected to be achieved?   Was there anything you think should have been done differently in terms of app implementation and support to parents? |
